# DIVA metabolomics: Differentiating vaccination status following viral challenge using metabolomic profiles

**DOI:** 10.1371/journal.pone.0194488

**Published:** 2018-04-05

**Authors:** Darren W. Gray, Michael D. Welsh, Fawad Mansoor, Simon Doherty, Olivier P. Chevallier, Christopher T. Elliott, Mark H. Mooney

**Affiliations:** 1 Institute for Global Food Security (IGFS), School of Biological Sciences, Queen’s University Belfast (QUB), Belfast, Northern Ireland, United Kingdom; 2 Veterinary Sciences Division (VSD), Agri-Food and Biosciences Institute (AFBI), Belfast, Northern Ireland, United Kingdom; University College Cork, IRELAND

## Abstract

Bovine Respiratory Disease (BRD) is a major source of economic loss within the agricultural industry. Vaccination against BRD-associated viruses does not offer complete immune protection and vaccine failure animals present potential routes for disease spread. Serological differentiation of infected from vaccinated animals (DIVA) is possible using antigen-deleted vaccines, but during virus outbreaks DIVA responses are masked by wild-type virus preventing accurate serodiagnosis. Previous work by the authors has established the potential for metabolomic profiling to reveal metabolites associated with systemic immune responses to vaccination. The current study builds on this work by demonstrating for the first time the potential to use plasma metabolite profiling to differentiate between vaccinated and non-vaccinated animals following infection-challenge. Male Holstein Friesian calves were intranasally vaccinated (Pfizer RISPOVAL^®^PI3+RSV) and subsequently challenged with Bovine Parainfluenza Virus type-3 (BPI3V) via nasal inoculation. Metabolomic plasma profiling revealed that viral challenge led to a shift in acquired plasma metabolite profiles from day 2 to 20 p.i., with 26 metabolites identified whose peak intensities were significantly different following viral challenge depending on vaccination status. Elevated levels of biliverdin and bilirubin and decreased 3-indolepropionic acid in non-vaccinated animals at day 6 p.i. may be associated with increased oxidative stress and reactive oxygen scavenging at periods of peak virus titre. During latter stages of infection, increased levels of N-[(3α,5β,12α)-3,12-dihydroxy-7,24-dioxocholan-24-yl]glycine and lysophosphatidycholine and decreased enterolactone in non-vaccinated animals may reflect suppression of innate immune response mechanisms and progression to adaptive immune responses. Levels of hexahydrohippurate were also shown to be significantly elevated in non-vaccinated animals from days 6 to 20 p.i. These findings demonstrate the potential of metabolomic profiling to identify plasma markers that can be employed in disease diagnostic applications to both differentially identify infected non-vaccinated animals during disease outbreaks and provide greater information on the health status of infected animals.

## Introduction

Bovine Respiratory Disease (BRD) is a multifactorial disease characteristic of a viral-bacterial synergistic infection with predisposition from environmental stressors [1]. The disease constitutes a major source of economic loss through mortality, clinical disease and the associated treatments and long lasting reduced growth performance of infected young stock [2, 3]. The annual cost of BRD is estimated at $1billion in the USA, with preventative measures contributing a further $3billion [4, 5]. Vaccines are commonly used for controlling BRD viral pathogens [6], but despite seasonal vaccination, animals can become infected with each new outbreak [7], maintaining the infection within the population. The viral pathogens associated with BRD [Bovine Parainfluenza Virus type-3 (BPI3V), Bovine Respiratory Syncytial Virus, Bovine Viral Diarrhoea Virus and Bovine Herpes Virus-1] impair immune responses in infected animals and damage the respiratory tract allowing the establishment of secondary infections, that may develop further into bacterial pneumonia [8]. However, vaccinated animals can successfully clear viral infections faster than non-vaccinated animals through immune memory response, reducing the associated viral tissue damage or impairment of immune functions preventing the establishment of secondary bacterial and mycoplasma infections [6]. During disease outbreaks, identification of unvaccinated animals at the early stages of infection could provide a window for effective treatment and facilitate the removal of animals that pose a greater risk of becoming infected and transmitting the infection to more susceptible juvenile stock. Furthermore, halting viral disease progression to more severe and costly secondary bacterial infections through the identification of vaccine failure animals during infection outbreaks would reduce the level of antibiotic use in the agricultural industry.

The only definitive method for successfully identifying vaccinated animals in the presence of an active viral infection is to determine the rate of viral shedding by virus isolation, cytokine/interleukin profiling or virus neutralization assay [9]. These types of analysis require repeated sampling, a period for seroconversion and are expensive compared to serology based ELISA, and are therefore not routinely employed during endemic viral infection outbreaks. Differentiating infected from vaccinated animals (DIVA) marker vaccines (e.g. a modified wild type virus with a gene deletion resulting in the absence of a particular diagnostic antigen) can be employed to differentiate vaccine antibody responses from that of wild type virus. Companion serology based tests rely on seroconversion, and upon exposure to wild type virus the antibody response to DIVA vaccines will be masked by that of the wild type virus. Vaccine DIVA functionality is often limited to large viruses with increased potential for gene deletion and removal of redundant expressed antigens. Therefore, for viruses with small genomes such as paramyxoviruses (e.g. BPI3V and Bovine Respiratory Syncytial Virus of the BRD complex) where gene deletion of neutralizing antigens may reduce vaccine efficacy, alternative approaches are required to provide DIVA functionality. One approach is to design molecular DIVA vaccines that contain a marker nucleotide sequence differing from the wild type virus that can be employed in combination with PCR-based molecular diagnostics to differentiate between vaccine and wild virus strains [10, 11]. Successful differentiation of vaccinated from non-vaccinated animals using this technique requires concurrent vaccination and infection [12, 13], with a narrow diagnostic window post-infection for detection of DIVA vaccine and viral genetic material. Furthermore, detection of vaccine genetic material only demonstrates exposure to the vaccine and not the successful generation of immune protection, limiting functionality in assessment of herd level immunity. Consequently, there is a clear need for alternative diagnostic methods that can assess efficacy of vaccines and vaccination status of animals exposed to BRD viral pathogens at the early stages of infection prior to seroconversion and which do not require repeated sampling. Additionally, the lower initial exposure rates to viral infections in field settings combined with variation in strain nucleotide sequences and short periods of virus secretion highlights the requirement for a DIVA approach with a long diagnostic window which is not strain specific.

A potential approach that can meet these needs is based on the application of metabolomics to identify metabolites or ‘small molecules’ in biological samples that are signatures that correlate or provide some evidence of immune protection. These metabolites are often the end stage products of biological processes and therefore provide an accurate representation of an organism’s homeostatic status at time of sampling [14, 15]. Metabolomic analysis of bio-fluids has provided new insights to the understanding of the patho-physiological processes involved in disease establishment, development and diagnosis [16–19]. Whilst metabolomics has had limited application in the field of veterinary research, several studies have demonstrated the potential of this technique in the prediction of BRD disease outcome [20], differentiation of stress from viral infection responses [21], and characteristic of immune responses following vaccination [22]. This study focuses specifically on BPI3V due to its endemnicity within cattle populations and absence of clinical symptoms which still predispose animals to more severe bacterial infections [23]. Due to its small genome and absence of non-redundant proteins suitable for removal in DIVA vaccines, BPI3V is an excellent model for assessing the potential of metabolomics to establish vaccination status in infected animals. The aims of the current study were therefore to assess the performance of Reverse Phase (RP) and Hydrophobic Interaction Liquid Chromatography (HILIC) separation methods for Ultra Performance Liquid Chromatography-Mass Spectrometry (UPLC-MS) metabolomic profiling of bovine plasma and identify plasma metabolomic markers capable of differentiating between vaccinated and non-vaccinated calves following intranasal challenge with BPI3V. This work for the first time reports the metabolomic responses following challenge with BPI3V and demonstrates how the application of metabolomic profiling may help overcome current limitations in DIVA diagnostics by identifying markers capable of differentiating between vaccinated and non-vaccinated animals, and importantly allow the development of better tools to assess the performance of vaccines.

## Results

### Clinical health assessment of experimental animals

Assessment of clinical findings of animals post BPI3V vaccination and challenge have been reported in detail previously [22]. Briefly, animals were healthy throughout the duration of the study with no clinical signs of disease in vaccinated or non-vaccinated study groups. Calves were sourced from respiratory disease free farms with no history of vaccination against BRD. Prior to the commencement of vaccination all calves tested seropositive for anti-BPI3V IgG. These residual levels of maternally derived anti-BPI3V immunoglobulin are in keeping with other studies employing calves of the same age [24]. At the commencement of vaccination there was no significant difference in anti-BPI3V IgG observed between treatment groups. Vaccination with RISPOVAL^®^PI3+RSV resulted in a significant increase in anti-BPI3V IgG with no significant increase in non-vaccinated controls. Following BPI3V challenge vaccinated calves had significantly higher plasma anti-BPI3V IgG relative to non-vaccinated animals. Post-BPI3V challenge, anti-BPI3V IgG remained elevated in vaccinated animals. Although no significant differences in anti-BPI3V IgG levels were observed in non-vaccinated animals at days 12–20 post-infection (p.i.), IgG was elevated in 2/3 animals relative to day 0 levels, with the remaining animal demonstrating elevated levels from days 14 to day 20 p.i. There was a significant increase in lymphocyte counts in non-vaccinated animals from day 0 to 5 p.i., and a significant decrease from days 5 to 12, with no significant variations observed in vaccinated animals. There were no significant differences in neutrophil counts between study groups at sampling points post-infection, however significant (p < 0.05) temporal variations were observed with a decrease from day 0 to 5 in non-vaccinated animals, and an increase from day 5 to 12 in both study groups. At day 6 p.i., 3 animals per group were prepared for gross post-mortem analysis. Two of 3 animals in the non-vaccinated group showed gross inflammatory lesions, with some peri-vascular cuffing consistent with pneumonia. No significant gross / histological abnormalities were detected in 3/3 of the vaccinated calves at 6 day p.i. at post-mortem.

### Evaluation of metabolomic methods for profiling of bovine plasma by RP and HILIC-UPLC-MS

**Fig 1:**
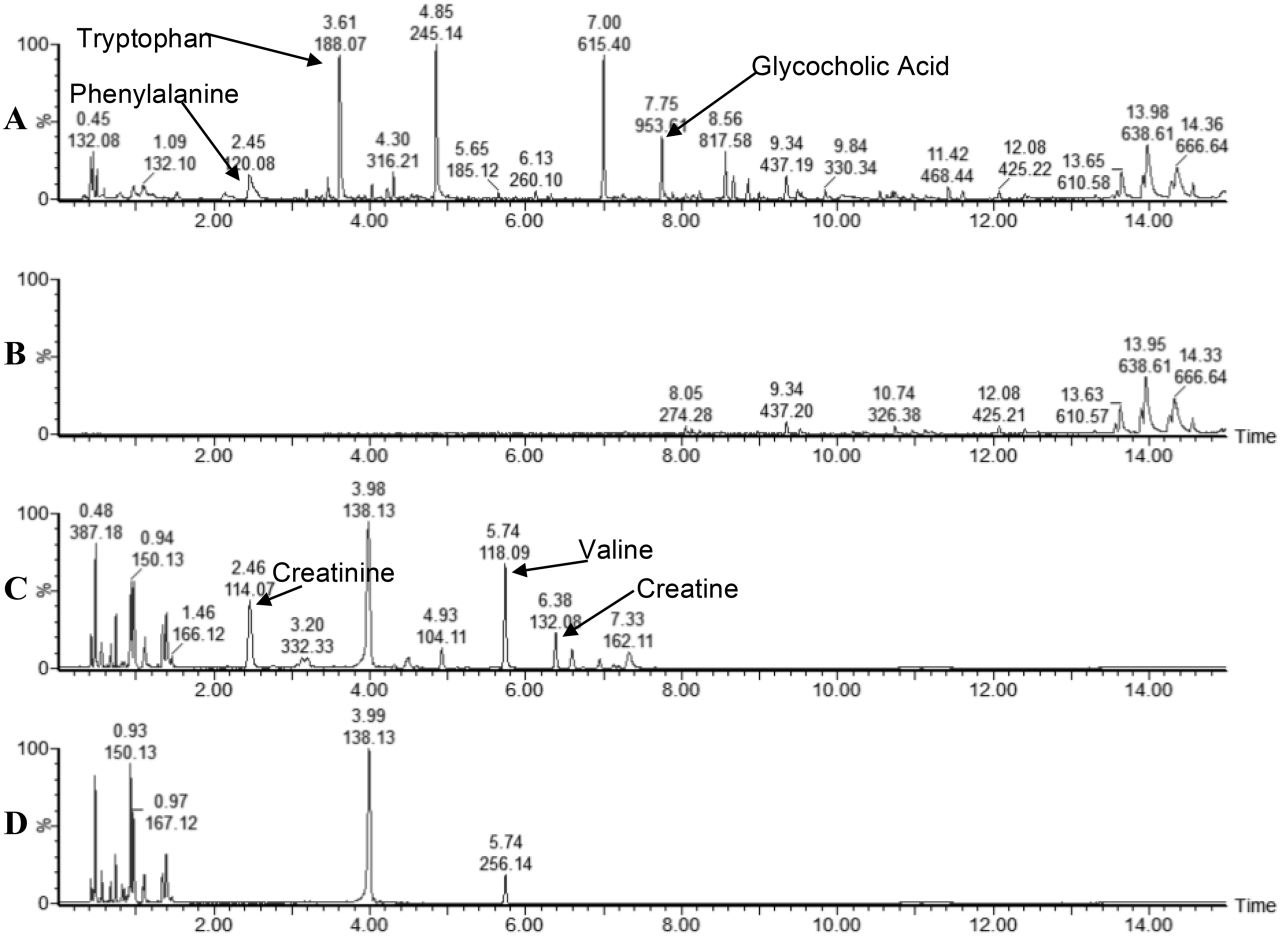
Baseline peak intensity chromatograms of RP and HILIC metabolomic profiled plasma at day 6 p.i. (A and C) Plasma samples analysed by RP and HILIC respectively, (B and D) control injections (extractions performed without plasma) analysed by RP and HILIC respectively. Base peak intensity for plasma analysed by RP was limited to 80,000 counts to reduce the influence of tryptophan (3.61 min). Peaks from reference compounds (confirmed using pure standards) are indicated, tryptophan, phenylalanine and glycocholic acid for RP, and creatinine, creatine and valine for HILIC.

**Fig 2:**
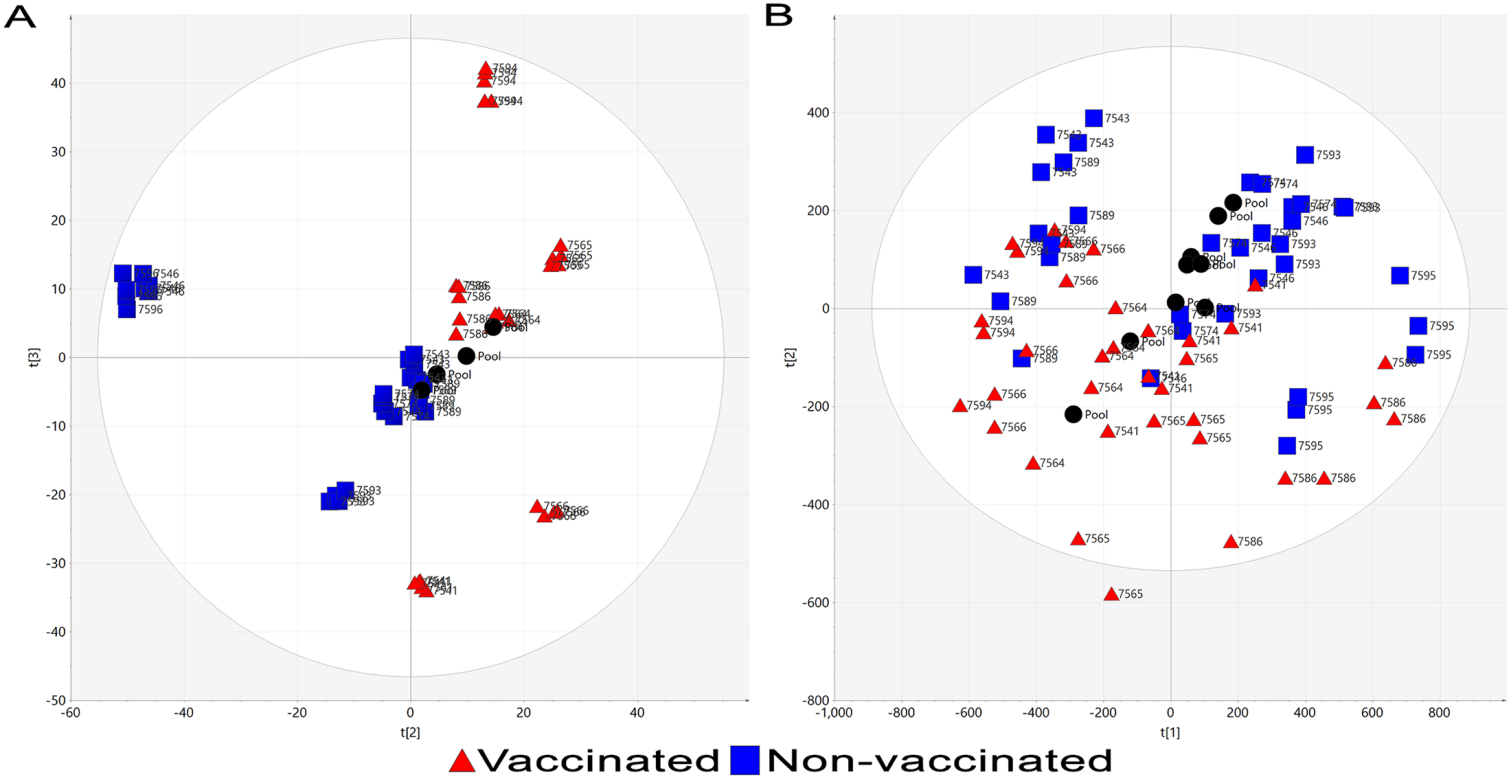
Unsupervised PCA scores plot of plasma from vaccinated and non-vaccinated calves at day 6 post-BPI3V challenge. PCA scores plot of day 6 p.i. plasma profiles acquired from vaccinated and non-vaccinated animals following (A) RP or (B) HILIC chromatographic separation.

Preliminary metabolomic analysis was performed to select a suitable method for the comprehensive profiling of metabolites within bovine plasma. RP and HILIC chromatographic methods were compared using day 6 p.i. samples (n = 6), which corresponds to peak viral titre [25]. Despite improved resolution of poorly retained hydrophobic compounds eluting between 1-2min by HILIC separation, increased chromatographic peaks corresponding to plasma derived compounds (relative to blank injections) were observed in RP acquired profiles (Fig 1A and 1B) relative to HILIC profiles (Fig 1C and 1D). Observable differences between plasma samples from vaccinated and non-vaccinated animals within chromatogram profiles were only present using RP (S1 Fig). Furthermore, upon data extraction and processing (metabolites with CV > 50% in QC pools), increased numbers of accurate mass retention time pairs (AMRTPs) were observed following RP (n = 1165) relative to HILIC (n = 538) separation of plasma. Unsupervised PCA analysis of plasma profiles acquired by RP-UPLC-MS resulted in clear separation between study groups when assessing principle components (PC) 1 and 2 (57.4% of systemic (R2X) variation within the dataset) as illustrated in Fig 2A. In contrast, HILIC-UPLC-MS metabolomic profiles facilitated only partial separation between study groups based on the PCA scores plot (PC1 and PC2, 35.6% of systemic (R2X) variation) (Fig 2B). Due to the small number of chromatographic peaks, fewer AMRTPs and poor unsupervised PCA separation of plasma profiles acquired following HILIC-UPLC-MS chromatography, RP-UPLC-MS was selected as the method of choice for metabolomic profiling of remaining study plasma samples.

### RP-UPLC-MS metabolomic profiling of bovine plasma

**Fig 3:**
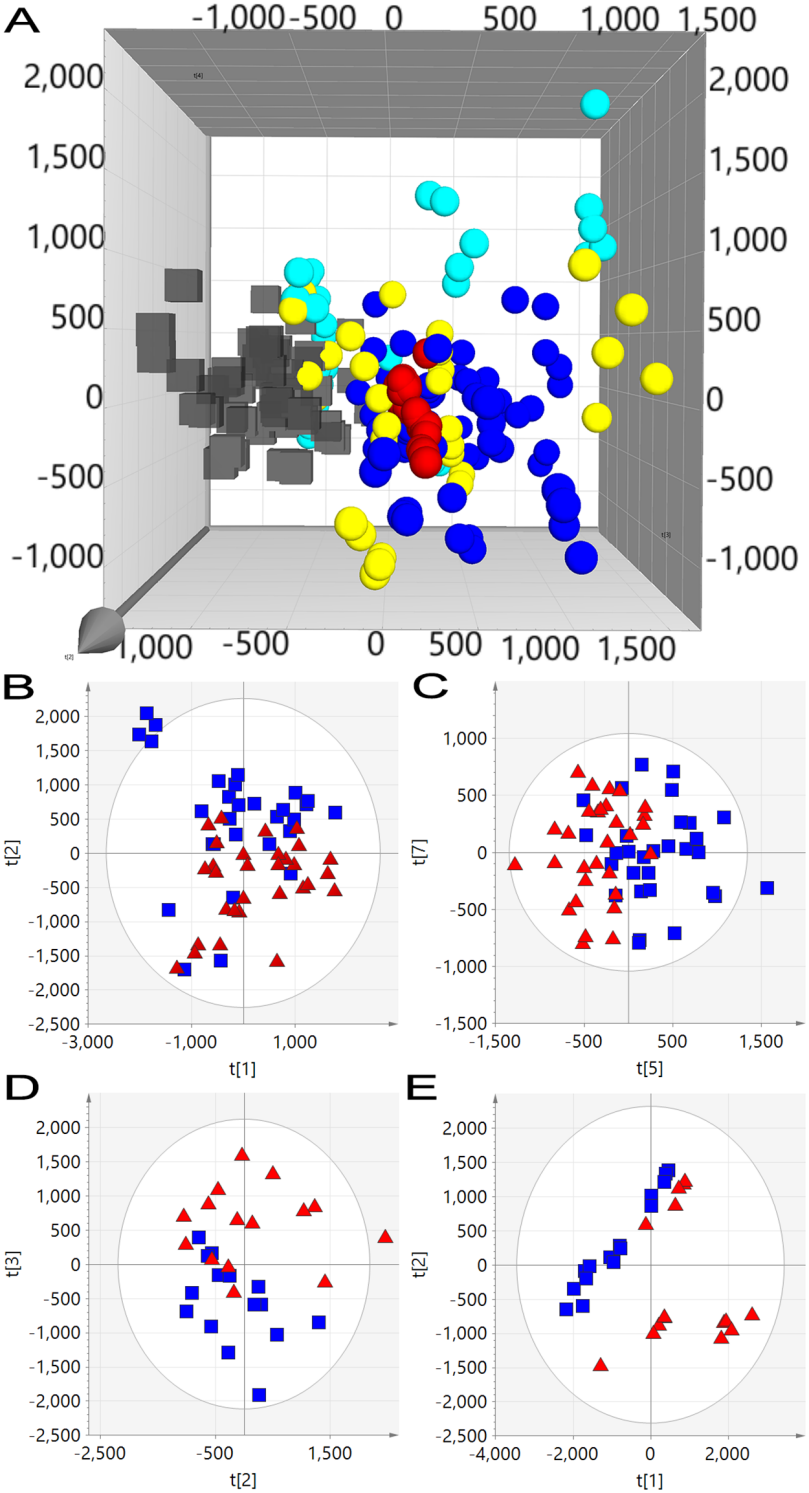
Unsupervised PCA scores plots of RP-UPLC-MS profiled plasma from vaccinated and non-vaccinated calves following BPI3V challenge. (A) PCA analysis of pre- (black cubes) and post-BPI3V challenge (spheres) plasma metabolite profiles from vaccinated and non-vaccinated animals. (Blue sphere) day 2 p.i., (red sphere) day 6 p.i., (yellow sphere) day 14 p.i. and (turquoise sphere) day 20 p.i. Quality control plasma pools are indicated with green snowflakes. PCA scores plots of plasma from vaccinated and non-vaccinated calves at (B) day 0, (C) day 2, (D) day 14 and (E) day 20 p.i.

Plasma samples from days 0, 2, 14 and 20 p.i. were extracted, analysed and combined with day 6 p.i. data for multivariate analysis. Mass accuracy and retention time deviation of reference compounds between analysis runs was excellent (S1 Table) and within Marker Lynx extraction parameters (0.02Da and 0.2min respectively) ensuring accurate peak matching. AMRTPs (n = 1593) with %CV less than 50% in inter-run quality control plasma pools were used to construct of PCA models (pre-filtered from 7690 AMRTPs extracted from raw data). The effect of BPI3V challenge on the metabolite profile of all animals irrespective of vaccination status, was illustrated in un-supervised PCA scores plot (Fig 3A) by separation in pre- (day 0 p.i.) and post-BPI3V challenge samples (days 2–20 p.i.) when observing PC2 and PC3 (14.1% R2X). The greatest separation was observed between day 0 pre- and day 2 p.i. post-challenge stages in vaccinated and non-vaccinated animals. Differentiation of vaccinated from non-vaccinated animals based on metabolite profiles following challenge with BPI3V was investigated using un-supervised (PCA) and supervised (OPLS-DA) multivariate analysis. Prior to BPI3V challenge metabolite profile variation between vaccinated and non-vaccinated animals was low, evidenced by no separation at day 0 (Fig 3B) when observing all PCs. With study progression to post-challenge stages, the variation between vaccinated and non-vaccinated animal metabolite profiles increased from partial separation at day 2 p.i. (Fig 3C, 9.45% R2X with PCs 5 and 7) to clear separation at days 6, 14 and 20, with 19% (Fig 2A), 32.1% (Fig 3D) and 51.1% (Fig 3E) variation (R2X) respectively in PCs 2 and 3.

### Selection of potential DIVA metabolite markers by supervised discriminant analysis

**Fig 4:**
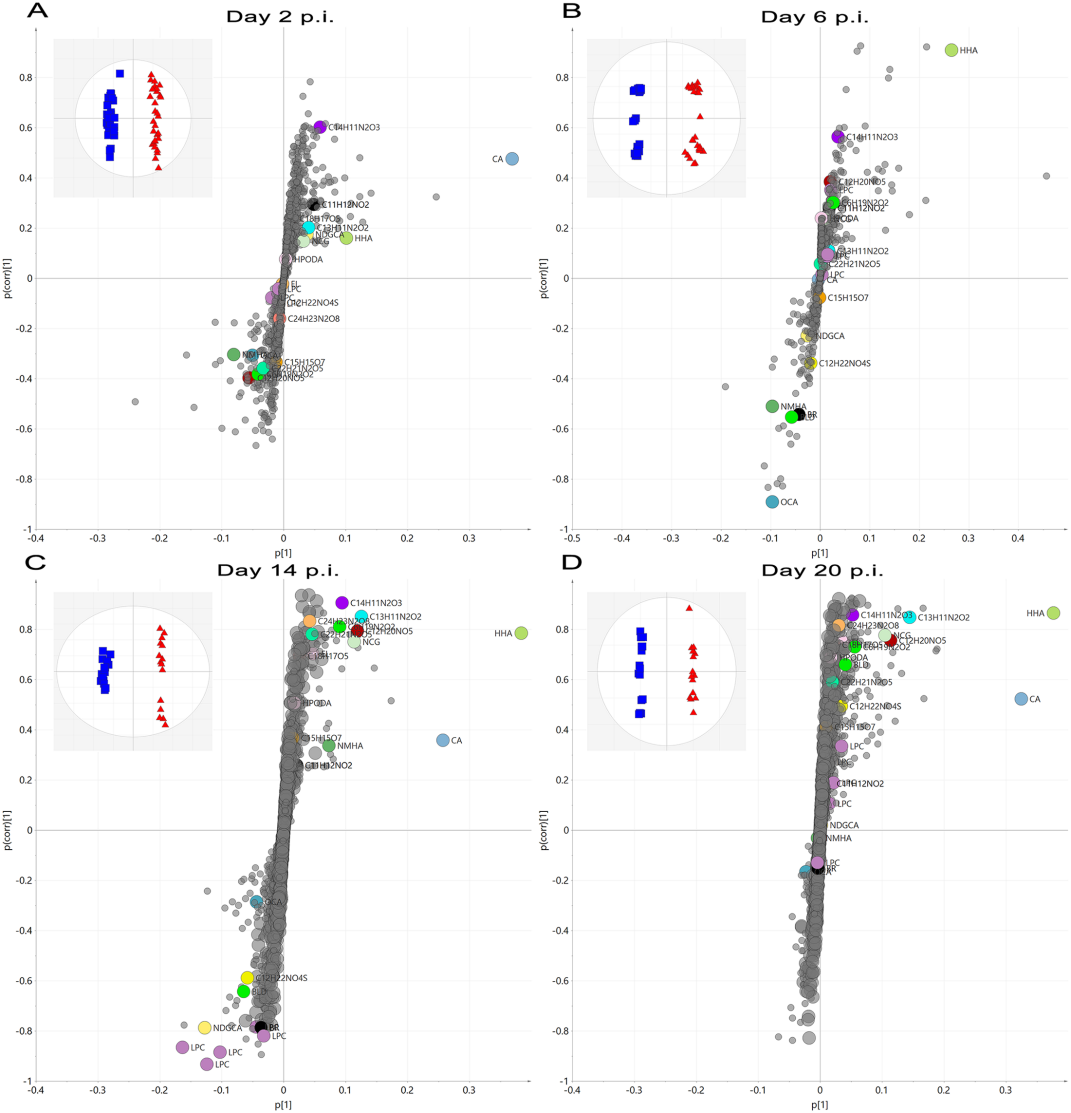
S-plots of supervised OPLS-DA analysis of RP-UPLC-MS profiled plasma metabolites from vaccinated and non-vaccinated calves following BPI3V challenge. Supervised OPLS-DA S-plot plots of plasma metabolite profiles from vaccinated and non-vaccinated calves at days 2, 6, 14 and 20 p.i. are illustrated in figures A-D respectively. The location of AMRTPs on the S-Plot is a combination of influence (p [1]) and reliability p(corr) [1] on the discrimination of study groups by OPLS-DA. Selected and identified vaccine response markers and AMRPTs selected are indicated on the S-Plot.

OPLS-DA analysis was employed for the selection of marker AMRTPs that could discriminate between vaccinated and non-vaccinated animals post-infection. Fig 4A–4D illustrates OPLS-DA score (inset) and S-plots for supervised discriminate analysis of plasma metabolite profiles from BPI3V infected vaccinated and non-vaccinated animals at days 2, 6, 14 and 20 p.i. respectively. The amount of variation responsible for differentiation of animals of different vaccination status (R2X) was 7.02%, 8.09%,12.7% and 20.4% in models generated for days 2, 6, 14 and 20 p.i. respectively, with excellent fit (R2Y > 98.5%) and good cross-validated prediction (Q2 > 95%). As OPLS-DA is known to over-fit, particularly in megavariate datasets where the number of variables are higher than the sample size we employed permutation and false discovery rate testing to reduce the chances of selecting false positives. Leave-one-out cross validation was performed to assess the performance of the models generated. Briefly, all technical replicates from a single biological sample (test sample) were removed and the remaining dataset employed to generate a predictive OPLS-DA model (vaccinated vs non-vaccinated). This predictive OPLS-DA model was employed to predict the vaccination treatment group of the test sample. This cross-validation was permeated until all biological samples had been assessed for treatment classification. The results (S2 Table) indicated that all biological replicates were accurately classified to their respective treatment groups upon cross validation prediction model testing. AMRTPs which contributed to class discrimination were selected on a criterion of a variable importance score (V.I.P.) score > 1 from OPLS-DA discriminate analysis. AMRTPs were further filtered to select only those with a fold change (FC) >1.5 and significant difference (ANOVA with bonferroni post-hoc test) p < 0.05 between study groups. Final selection of AMRTPs was performed by assessment of raw mass spectrometric data to determine those with good peak shape and consistent peak intensity (height) in replicate injections. 383 unique potential markers combined from all OPLS-DA analysis (44 at day 2 p.i., 152 at day 6 p.i., 128 at day 14 p.i., and 91 at day 20 p.i.) were selected for further refinement to remove fragments and adducts for parent ion identification.

### Database and *in silico* identification of plasma metabolites with DIVA marker potential

The selected panel of 383 unique AMRTPs (S3 Table) differentiating animals of different vaccination status at various time-points post-BPI3V challenge were deconvoluted to identify parent ion mass, adducts and low energy fragments using low and high energy data (Function 1 and 2 respectively), yielding 26 parent ions for elemental composition determination. Non-parametric Mann Whitney was also employed on the selected panel of metabolites from day 0, 2 and 6 p.i. time points, with all markers attaining significance levels within thresholds comparative with ANOVA. The false discovery rate was calculated from the CV (50%) filtered dataset via the Benjamini-Hochberg procedure with a threshold of 20%. All selected markers passed this threshold and met further selection criteria (V.I.P. score > 1 and FC > 1.5) were selected as potential markers. Retention times and accurate masses of potential markers post-BPI3V challenge (days 2, 6, 14 and 20 p.i.) are shown in Table 1. Four metabolites (Hexahydrohippurate (HHA), Biliverdin (BLD), Cholic Acid (CA) and Lysophosphatidycholine (LPC) (16:0/0:0)) were confirmed using pure analytical standards, and the remaining 17 assigned putative identifications based on spectral matching against Metlin or HMDB databases (N-Methylhippuric Acid (NMHA), N-(Cyclohex-1-en-1-ylcarbonyl)glycine (NCG), Enterolactone (EL), 3-Indolepropionic acid (3IPA), Bilirubin (BR), 3-Oxocholic Acid (OCA), Hydroperoxyoctaeca-9,12-dienoic acid (HPODE), (N-[(3α,5β,12α)-3,12-Dihydroxy-7,24-dioxocholan-24-yl]glycine (NDGCA), Lysophosphatidylcholine (LPC) LPC(18:2(9Z,12Z)), LPC(0:0/16:0), LPC(18:0/0:0), LPC(18:1(11Z)/0:0) and LPC(17:0/0:0)). Significant differences in the plasma peak intensities of these metabolites between vaccinated and non-vaccinated animals are highlighted in Table 1. S2 Fig highlights peak intensity of the identified metabolite markers for individual animals throughout the study. HHA, NCG and NMHA exhibited analogous fragmentation and adduct formation pattern (S3 Fig), indicating similar molecular structure, and a significant correlation (p < 0.001) between the levels of HHA and NCG observed (R^2^ = 0.98). Plasma CA levels were found to increase significantly only in vaccinated animals over day 0 to 20 p.i. (FC = 2.77) with no significant changes evident in non-vaccinated animal plasma.

**Table 1:** Metabolomic markers identified by RP-UPLC-MS/MS analysis of plasma.

AMRPT I.D. (RT_m/z)	Elemental Composition (M+H)	% Fit	Mass Error (mDa)	Fold change post BPI3V challenge (Non-vaccinated vs vaccinated)				Identity	Database identifier
				Day 2 p.i.	Day 6 p.i.	Day 14 p.i.	Day 20 p.i.		
**0.42_151.1442**	**C6 H19 N2 O2**	100	-0.3	1.57	-2.15	-4.74 *	-2.33	Unknown	N/A
**3.00_393.1432**	**C22 H21 N2 O5**	13	-0.7	1.97	1.84	-3.36 **	-2.38	Unknown	N/A
**4.57_194.0819**	**C10 H12 N O3**	100	-0.2	1.13	2.46*	-1.17	1.11	N-Methylhippuric Acid (NMHA)	99223 –PubChem
**4.57_227.0822**	**C13 H11 N2 O2**	100	-0.4	1.02	-1.12	-3.21 **	-6.68 *	Unknown	N/A
**4.85_184.0976**	**C9 H14 N O3**	100	0.4	1.06	-5.52 *	-3.60	-10.80	N-(cyclohex-1-en-1-ylcarbonyl)glycine (NCG)	149048 –PubChem
**4.98_186.1132**	**C9 H16 N O3**	100	0.1	1.12	-9.46 ***	-2.05 *	-4.64 *	Hexahydrohippurate (HHA) ^a^	147412 –PubChem
**4.99_276.1268**	**C12 H22 N O4 S**	60	-0.7	1.28	3.46 **	1.54	-1.38	Unknown	N/A
**5.17_258.1342**	**C12 H20 N O5**	97	0	1.66	-6.21 ***	-3.56 *	-7.04	Unknown	N/A
**5.27_299.1286**	**C18 H19 O4**	51	0.2	1.20	ND	-1.91	-2.35 *	Enterolactone (EL)	10685477 –PubChem
**5.61_313.1077**	**C18 H17 O5**	70	0.9	1.04	2.09	-1.76 *	-2.90 *	Unknown	N/A
**6.10_467.1461**	**C24 H23 N2 O8**	73	-0.5	1.42	ND	-10.69 *	-8.72	Unknown	N/A
**6.40_190.0869**	**C11 H12 N O2**	100	-0.4	-1.17	-2.40*	-1.16	-1.49	3-indolepropionic acid (3IPA)	3744 –PubChem
**7.10_464.3010**	**C26 H42 N O6**	46	0.5	-1.32	1.30	1.74 *	-1.82	N-[(3α,5β,12α)-3,12-Dihydroxy-7,24-dioxocholan-24-yl]glycine (NDGCA)	8429342 –Chemspider
**7.24_255.0771**	**C14 H11 N2 O3**	99	-0.5	-1.17	-2.69 *	-4.28 *	-4.42 *	Unknown	N/A
**7.40_585.2708**	**C33 H37 N4 O6**	89	0.5	-2.88	3.25 *	4.88 *	2.60	Bilirubin (BR)	5280352 –PubChem
**7.82_313.2377**	**C18 H33 O4**	95	0.4	-1.02	ND	-2.38	-2.69 *	(9Z,12Z)-(8R)-8-Hydroperoxyoctadeca-9,12-dienoic acid (HPODE)	C14831—KEGG
**7.92_389.2691**	**C24 H37 O4**	88	1	-1.07	3.4 ***	1.24	1.14	3-oxocholic acid -H2O (OCA)	5283956 –PubChem
**8.51_583.2611**	**C33 H35 N4 O6**	27	1.3	1.24	2.86 *	1.58	-1.26	Biliverdin (BLD) ^a^	5280353 –PubChem
**8.52_817.5824**	**2(C24 H40 O5)H**	75	0.8	-1.78	-1.02	-1.46	-2.72 *	Cholic Acid (Dimer—no parent) (CA) ^a^	221493—PubChem
**10.17_520.3402**	**C26 H51 N O7 P**	13	0.8	1.14	-1.29	5.61 *	1.01	LysoPC (LPC) (18:2(9Z,12Z))	C04230 –KEGG
**10.29_496.3398**	**C24 H51 N O7 P**	77	0.2	-1.10	ND	3.83 *	1.32	LysoPC (LPC) (0:0/16:0)	53478599 –PubChem
**10.49_496.3400**	**C24 H51 N O7 P**	4	-1.3	-1.01	1.03	2.99 *	-1.09	LysoPC (LPC) (16:0/0:0) ^a^	460602 –PubChem
**10.57_546.3559**	**C28 H53 N O7 P**	46	1.7	1.38	1.16	9.35 *	2.00	LysoPC (LPC) (18:0/0:0)	497299—PubChem
**10.79_522.3479**	**C26 H53 N O7 P**	97	0	1.10	1.26	3.95 ***	1.03	LysoPC (LPC) (18:1(11Z)/0:0)	53480465—PubChem
**11.02_510.3560**	**C25 H53 N O7 P**	43	0.2	1.17	7.89	2.23 *	-1.41	LysoPC (LPC) (17:0/0:0)	24779463 –PubChem

Markers were selected on the criteria FC > 1.5, p < 0.05 and V.I.P. score > 1. Fold change (FC) in plasma peak intensity in vaccinated animals relative to non-vaccinated is indicated (p < 0.05 *, p < 0.01 **and p < 0.001 ***).

^a^—Identified using extracted ion mass spectrum (MS) (low and high energy ramp) and retention time under matching against analytical standards under identical experimental conditions.

ND—Not detected

N/A—No database identifier available.

## Discussion

This is the first study to report the potential to differentiate between infected animals with differing vaccination status through the use of metabolomic profiling techniques (DIVA metabolomics). Previous work [22] by the authors has demonstrated that metabolomics can identify metabolites associated with immune responses to vaccination, and the current study has advanced this concept by applying metabolomics analysis of plasma to identify unique metabolite marker profiles that are capable of distinguishing between vaccinated and non-vaccinated animals following infection. Calves vaccinated (RISPOVAL^®^PI3+RSV) and infection-challenged (BPI3V) demonstrated a significantly stimulated anti-BPI3V IgG post-vaccination response which remained elevated post-challenge. Lung histology and haematology confirmed that the BPI3V inoculum employed for viral challenge was sufficient to induce some evident respiratory pathology in non-vaccinated animals, with an absence in vaccinated animals. The subsequent challenge in primed vaccinated animals also resulted in maintenance of pre-challenge elevated anti-BPI3V IgG (indicating response to inoculum), with absence of respiratory pathology. Following preliminary analysis of a subset of samples (day 6 p.i.), RP-UPLC-MS analysis demonstrated increased capacity to profile bovine plasma metabolites relative to HILIC-based chromatographic separation and was subsequently used for extensive metabolomic plasma profiling. Unsupervised PCA analysis of acquired metabolomic profiles of pre- and post-challenge plasma revealed clear and distinguishable variation in plasma metabolites between vaccinated and non-vaccinated animals. Considering that elevated anti-BPI3V IgG typically occurs at 2 weeks post-challenge [6, 26], variation in plasma metabolite profiles as early as day 2 p.i. illustrates the capacity of metabolomic profiling methods to detect changes stimulated by immune responses at early post-infection phases.

Supervised multivariate discriminant analysis of plasma metabolomic profiles yielded 383 potential metabolites (AMRTPs) that were significantly different in the plasma of vaccinated compared to non-vaccinated animals following BPI3V challenge. Following de-convolution (with removal of adducts and fragment ions), 26 parent metabolite ions were identified and shown to be present at different levels within plasma from vaccinated and non-vaccinated animals from days 6–20 p.i. Despite no significant differences in the parent ion intensity between treatment groups at day 2 p.i. plasma metabolite profiles differed between vaccinated and non-vaccinated animals through supervised OPSL-DA. Identities of 17 of these 26 parent metabolites were revealed via database searching, *in silco* fragmentation and spectral matching.

Divergent plasma metabolite profiles have previously [22] been reported following vaccination, with altered metabolites shown to be associated with primary or secondary immune responses to vaccination. The metabolomic profiling performed here in this study on post-BPI3V challenge acquired samples, has identified a unique panel of plasma metabolites which differ between vaccinated and non-vaccinated animals, and significantly are involved in recognised immune response mechanisms. At day 6 p.i., increased biliverdin (FC = 2.86), bilirubin (FC = 3.25) and decreased 3-indolepropionic acid (FC = -2.40) levels were observed in plasma of non-vaccinated animals. Biliverdin is degraded from heme by heme-oxygenases, and is further reduced to bilirubin by bilirubin reductase [27]. Heme-oxygenase-1 exerts anti-inflammatory effects as demonstrated by reduced tumour necrosis factor alpha release in lipopolysaccharide-stimulated macrophages [28]. 3-Indolepropionic acid is a reactive oxygen species scavenger [29], produced in the microbiome [30]. Phagocyte activation by RNA viruses results in both increased reactive oxygen species release and the production of pro-oxidant cytokines (tumour necrosis factor alpha and interleukin-1) which promote iron uptake by the mononuclear phagocyte system [31, 32]. Decreasing plasma 3-indolepropionic acid levels in concert with elevated biliverdin and bilirubin levels (via heme-oxygenase-1 action) in non-vaccinated animals at day 6 p.i. maybe indicative of 3-indolepropionic acid scavenging of reactive oxygen species produced by phagocytes, or increased downstream ROS production from cytokine signalling.

Fluctuations in the levels of a number of bile acids (3-oxocholic acid, cholic acid and NDGCA) were observed in plasma of animals at various stages in the study. 3-oxocholic acid and NDGCA were found to be significantly increased (FC = 3.4 and 1.74 respectively) in the plasma of non-vaccinated animals at day 6 and 14 p.i respectively, whereas cholic acid was significantly higher in vaccinated animals (FC = -2.72) at day 20 p.i. Altered plasma bile acid profiles at distinct study phases suggest that bile acid metabolism and conjugation is associated with different immune response mechanisms. Bile acid driven farnesoid X receptor activation (expressed in pulmonary endothelial cells [33, 34]) enables a regulatory immune response as demonstrated by dendritic cell modulation [35], Natural killer T-cell inhibition, reduced pro-inflammatory osteopontin production [36] and reduced lung permeability, suppressing leukocyte movement to sites of tissue inflammation [37]. When transported into cells NDGCA is a potent farnesoid X receptor activator [38] and elevated NDGCA at day 14 in non-vaccinated animals suggests an association with the switching and/or suppression of innate inflammatory responses towards adaptive immune responses. Cholic acid, the primary metabolite of cholesterol, was found to significantly increase from day 0 to 20 p.i. in vaccinated animals. Cholesterol, a component of lymphocyte lipid rafts, supports B- and T-cell receptor signalling [39]. Reverse cholesterol transport is associated with immunosuppression via reduction in B- and T-cell receptor signalling, lymphocyte activation and proliferation [40, 41], and elevated cholic acid may indicate increased cholesterol metabolism via reverse cholesterol transport in proliferating lymphocytes, down-regulating the secondary immune response to viral challenge during later stages of infection (day 20 p.i.).

Lysophosphatidylcholine produced from phosphatidylcholine [42–44] stimulates dendritic cell maturation through the action of a G-protein-coupled receptor with further ability to stimulate interleukin-2 and interferon-γ production in T cells [45] indicating a role in innate-to-adaptive immune response progression. Plasma levels of six lysophosphatidylcholine derivatives were significantly up-regulated (FC > 2.23) in non-vaccinated animals at day 14 p.i. Significantly higher plasma levels suggest involvement in systemic immune responses to primary BPI3V antigen stimulation with dendritic cell recruitment to lymph nodes and subsequent dendritic cell driven T-cell activation. Decreased plasma peak intensity of enterolactone (FC = -2.35), a lignan metabolite formed in ruminants through the action of colonic bacteria on Secoisolariciresinol Diglucoside [46], was observed in non-vaccinated animals at days 14 and 20 p.i. enterolactone crosses the intestinal barrier and exerts anti-inflammatory functions by suppressing nuclear factor-κB signalling, tumour necrosis factor alpha production [47] and interleukin-1β release [48]. As enterolactone is dietary derived it cannot be readily replenished or up-regulated during periods of high demand, therefore decreased enterolactone in non-vaccinated animals during the latter stages of BPI3V infection (days 14 and 20 post-challenge) may reflect its metabolism to induce anti-inflammatory effects associated with the transition of acute to adaptive immune responses.

From a diagnostic perspective, those plasma metabolite markers found to be altered at early stages of infection prior to sero-conversion and which persist to the later stages of infection are of particular interest. Hexahydrohippurate, despite not showing significantly altered plasma levels until day 6 p.i., remained elevated in vaccinated animals (FC > -2.05) for the duration of the study. Significantly lower levels of N-methylhippuric acid (FC = 2.46) and higher N-(cyclohex-1-en-1-ylcarbonyl)glycine (FC = -5.52) were also observed in vaccinated calves at day 6 p.i. N-(cyclohex-1-en-1-ylcarbonyl)glycine, N-methylhippuric acid and hexahydrohippurate are formed through the action of glycine N-acyltransferase on cyclohexanecarboxy-CoA, cyclohexene-1-carboxyl-CoA or benzoyl-CoA, produced during shikimate and phenylalanine metabolism [30, 49–52]. With no significant variation in the plasma levels of phenylalanine (which would attribute post-BPI3V challenge acyl-glycine conjugate to dietary variations), elevated hexahydrohippurate and N-(cyclohex-1-en-1-ylcarbonyl)glycine levels may be a consequence of an increased demand for CoA in the liver due to the need to free CoA otherwise sequestered in cyclohexanecarboxy-CoA or cyclohexene-1-carboxyl-CoA. Increased CoA demand may therefore be associated with increased rate of B- and T-cell proliferation and antibody production following an adaptive memory response to BPI3V challenge (as illustrated previously by post-parental immunization [22]). Assessment of hexahydrohippurate concentrations in plasma could allow for differentiation of vaccination status in infected calves with a wider diagnostic window to that observable currently through monitoring differences in anti-BPI3V IgG levels. Hexahydrohippurate occurs at high abundance in plasma offering potential for use as a realistic marker that could be measured using on-site based testing methods.

In conclusion, this study highlights the potential of untargeted UPLC-MS metabolomics to differentiate the vaccination statuses of virus challenged animals (i.e. DIVA Metabolomics). The differential pre-challenge immune status of vaccinated and non-vaccinated animals resulted in divergent plasma metabolite profiles following BPI3V challenge, evident as early as 2 days p.i., with increasing variation from time post challenge. The metabolites identified were associated with immune cell regulation mechanisms, including T/B-cell proliferation and phagocyte activation and maturation. The wide diagnostic window for hexahydrohippurate combined with metabolite markers altered at distinct time periods associated with specific immune response mechanism (e.g. LysoPC, biliverdin, bilirubin or 3-indolepropionic acid), could find application in staging of infection. The effectiveness of these efforts is reflected in the large magnitude fold-change and low intra-group variation of statistically significantly metabolites identified at days 14 and 20 p.i. Similar to cytokine profiling, metabolomic based diagnostics are unlikely to match the pathogen specificity of molecular and serological testing but instead provide greater information on physiological health status, with future disease diagnostics potentially employing multiple methods to improve disease management decisions. A limitation of this study, as with many other biomarker discovery investigations involving large bovine animals, is the small cohort size (i.e. n = 6 per group; n = 3 at days 14 and 21 p.i.) which can be incorporated effectively into experimental groups. This potentially may impact on how findings from resulting analysis can be accurately translated to that observed within the wider more variable herd population. To mitigate against such issues, animals were extensively screened with regards to health and maternal antibody status prior to study group inclusion, and post-metabolomics profiling, stringent metabolite selection criteria (FC > 1.5; p < 0.05; V.I.P. score > 1; 20% false discovery rate) were applied to select only the most robust metabolites as marker candidates. As such, further investigation using a larger, more comprehensive sample set (differing sexes, breeds and ages of animals) with alternative routes of vaccination, vaccination failure (degradation or immunosuppression) and challenge (with multiple pathogens) may determine a panel or ‘fingerprint’ of metabolites with greater diagnostic specificity. A number of unidentified markers showed promising expression profiles and as metabolomics is still a relatively new field, lacking the level of database curation compared to genomics and proteomics for marker identification, these markers may be identified in the future. Further studies are required to expand upon this initial proof-of-concept to determine if the observed DIVA metabolite markers are robust. Importantly these studies have identified potential markers that would also be of benefit in screening and assessing new vaccine formulations and allow identification of trails indicative of protective immune responses.

## Materials and methods

### Chemicals and reagents

HPLC grade acetone and analytical standards were purchased from Sigma Aldrich (Dorset, UK). LC-MS grade acetonitrile, water, methanol and chloroform were purchased from Fisher Scientific (Loughborough, UK).

### Ethics statement

All animal studies were carried out in accordance with the UK Animals (Scientific Procedures) Act 1986 and with the approval of the Agri-Food and Biosciences Institute Northern Ireland Ethical Review Committee.

### Experimental design and sample collection

Calves were vaccinated with RISPOVAL^®^PI3+RSV as previously reported [22]. Briefly, 12 male Holstein Friesian calves aged between 20 and 25 weeks were sourced commercially from farms with no history of prior respiratory disease outbreaks. The calves had no prior vaccination for BRD and were clinically examined and declared fit for the study on day 0 by the Named Veterinary Surgeon. Claves were divided into two study groups (n = 6) and assigned as non-vaccinated and vaccinated calves. Vaccinated calves were treated with Pfizer RISPOVAL^®^PI3+RSV intranasal vaccine (designated vaccinated animals) as per manufacturer’s instructions, and non-vaccinated calves treated with empty poly-(lactic-co-glycolic) acid nanoparticles (designated non-vaccinated) prepared using standard double emulsification solvent evaporation technique (w/o/w) [53]. Calves received two dosages of vaccine formulation at 70 and 35 days prior to intranasal BPI3V challenge (post-infection = p.i.) (inoculation with 2 mL of virus suspension (TCID_50_ of 10^6.78^/mL) per nostril). Calves were screened weekly following vaccination and at days 0, 5, 12, 14 and 20 post-BPI3V infection (p.i.) for the presence of BPI3V IgG in blood serum using Svanovir-PI3V-Ab kit (Boehringer Ingelheim Svanovir, Uppsala, Sweden) as per manufacturer’s instructions. At day 6 p.i. 3 animals per group were sacrificed for viral isolation and histology after sampling. Blood samples drawn via jugular venepuncture at days 0, 2, 6, 14 and 20 p.i. into 6ml plastic K3 EDTA Vacuette tubes (Greiner bio-one, Stroudwater, UK) were processed at random to platelet poor plasma via a double centrifugation method [54] optimized for metabolomic analysis within 2 hours of initial blood drawing and plasma stored at -80°C prior to use.

### Plasma preparation for Reverse Phase (RP) Ultra Performance Liquid Chromatography Mass Spectrometry (UPLC-MS) metabolomic profiling

Samples processing order was randomized to negate processing bias on sample metabolite profiles. 400 μL of plasma was added to 1.6 mL of ice cold acetone, vortexed for 30 sec and placed on ice for 15 min. The sample was then deproteinated by centrifugation at 15,000*g* at 4°C for 15 min. 1.6 mL of supernatant was removed and dried under nitrogen for 45 min at 40°C using TurboVap LV (Caliper Life Sciences, Hopkinton, USA). Resulting residue was reconstituted in 500 μL of ultra-pure H_2_O and Liquid/Liquid extraction of lipids performed by addition of 500 μL of ice-cold methanol:chloroform (1:1 v/v) and vortexing for 30 sec followed by centrifugation at 16,000*g* at 4°C for 15 min. Liquid/Liquid extraction was repeated and after centrifugation 900 μL of the aqueous layer was removed and dried under nitrogen. The residue was reconstituted in 150 μL ultra-pure H_2_O and filtered by centrifugation at 10,000*g*, 4°C using 0.22μm Costar Spin-X^®^ Centrifuge Tube Filter for 5 mins.

### Plasma preparation for Hydrophobic Liquid Interaction Chromatography (HILIC) UPLC-MS metabolomic profiling

100 μL of plasma was added to a well in an Ostro protein precipitation and phospholipid removal plate (Waters Corporation, Milford, MA, USA). 100μL of 1% formic acid/acetonitrile (v/v) was added to the sample well, the plate shaken for 30 sec and extracted metabolites drawn through under vacuum into a 96 well 2 mL collection plate.

### Metabolomic profiling of bovine plasma by RP-UPLC-MS

RP-UPLC-MS analysis was performed using an Acquity UPLC system coupled to a XEVO G2 Q-Tof (Waters Corporation, Milford, MA, USA). A test mix of Acetominophen, Sulfaguanidine, Sulfadimethoxine, Val-Tyr-Val, Verapmil, Terfenadine, Leucine-Enkephalin, Reserpine and Erythromycin was injected to ensure calibration of mass spectrometer mass accuracy and UPLC performance prior to analysis. Pooled samples (comprising a 10μl aliquot from all study samples) were injected 5 times before the start of each run for column conditioning [55] and intermittently throughout the run to validate instrument performance. The run-order of samples entering the mass spectrometer was constructed using a randomized sample list comprising 5 technical replicates per biological sample. No other samples were injected during the analysis run. 8 μL of prepared sample extracts were injected onto an Acquity UPLC HSS-T3 column (100 mm x 2.1 mm i.d., 1.8 μm; Waters Corporation, Milford, MA, USA). Column and autosampler temperature were maintained at 50°C and 10°C respectively. Chromatographic separation was carried out at a flow rate of 600 μL/min with mobile phase consisting of 99.9% H2O/0.1% formic acid (A) and 99.9% acetonitrile/0.1% formic acid (B). The elution gradient was as follows: 0–2 min isocratic at 1% of B, 2–14.5 min linear gradient form 1–100% of B, 14.5–17.5 min isocratic at 100% of B, and finally 17.5–20 min linear gradient at 100–1% of B. Mass spectrometry was performed in positive-ion mode (ESI+) with the capillary voltage set to 1500 V and the sampling cone voltage 30V. The desolvation and cone gas flows were set at 750 L/h and 100 L/h respectively. Source and desolvation temperatures were 120°C and 400°C respectively. Leucine Enkephalin ([M+H]^+^ = 278.1141 Da, and [M+H]^+^ = 556.2771 Da) was used for accurate lockmass calibration during data acquisition. Lockmass acquisition setting were: 0.5 sec scan time, 30 sec interval, 3 scan average, mass window +/- 0.5Da. Centroid data were acquired in positive mode using resolution mode. Collision energy was only applied on function 2, with ramping between 15 eV and 30 eV.

### Metabolomic profiling of bovine plasma by HILIC-UPLC-MS

For HILIC-UPLC-MS/MS analysis the test mix was cytosine, O-acetyl-L-carnitine and L-valine. Pooled samples (comprising a 10μl aliquot from all study samples) were injected 5 times before the start of each run for column conditioning [55] and intermittently throughout the run to validate instrument performance. The run-order of samples entering the mass spectrometer was constructed using a randomized sample list comprising 5 technical replicates per biological sample. No other samples were injected during the analysis run. 1 μL of prepared sample extracts were injected onto an Acquity UPLC BEH HILIC column (2.1 mm x 100 mm i.d., 1.7 μm; Waters Corporation, Milford, MA, USA). Column and autosampler temperature were maintained at 45°C and 6°C respectively. Chromatographic separation was carried out at a flow rate of 600 μL/min with mobile phase consisting of 10 mM ammonium formate pH 3.3(A) and acetonitrile with 0.2% formic acid (B). The elution gradient was as follows: 0–2 min isocratic at 5% of A, 2–10 min linear gradient form 5–30% of A, 10–11 min linear gradient from 30–90% of A, 11–12 min isocratic at 90% of A, 12–12.5 min linear gradient from 90–5% of A, 12.5–16 min isocratic at 5% of A. Mass spectrometry was performed using a Waters Xevo G2 Q-Tof (Milford, MA) operating in positive-ion mode (ESI+) with the capillary voltage set to 300V and the sampling cone voltage 20V. The desolvation and cone gas flows were set at 700L/h and 5L/h respectively. Source and desolvation temperatures were 120°C and 450°C respectively. Leucine Enkephalin was used for accurate lockmass calibration during data acquisition, with acquisition settings the same as with RP-UPLC-MS analysis. Centroid data were acquired in positive mode using resolution mode. Collision energy was only applied on function 2, with ramping between 20V and 35V.

### UPLC-MS metabolomics data processing

Total Ion Count (TIC) chromatograms and spectra were acquired with MassLynx version 4.1 (Waters Corporation, Milford, MA, USA) in centroid format and metabolite data was processed using the MarkerLynx software. The MarkerLynx method for data extraction and de-convolution was as follows. Ions were extracted from function 1 data using peak detection analysis of retention time window 0.30–14.50min, with a mass range of 100Da to 1200Da. The XIC window for data collection was 0.2Da and apex peak tracking parameters were set to automatic with no smoothing. Data collection parameters consisted of an intensity threshold (counts) of 500, a mass window of 0.02Da with a retention time window of 0.20min. A noise elimination level of 6 was applied and isotopes were removed. Peak heights for extracted ions were normalized against the total peak height of all extracted ions and standardized to a total ion count of 10,000. The results were exported in .csv format as a two dimensional data table in which rows and columns respectively represented analysed samples and the relative normalized peak heights of each detected mass spectrometric signal, i.e. as an Accurate Mass (m/z) and Retention Time (min) Pair (AMRTP). Extracted and processed data submitted for downstream multivariate and statistical analysis for metabolite marker selection can be found in supplementary data (S4 Table).

Temporal changes in BPI3V antibody titre were analysed using two-tailed paired T-test, and significant differences between treatment groups at sampling stages was assessed using two-tailed heteroscedastic T-test. SIMCA-P+ version 13.0 (Umetrics, Sweden) was used for multivariate metabolite marker selection. The dataset was pre-filtered to exclude AMRTPs with coefficient of variation greater than 50% in inter-run quality control pools (generated from equal aliquots of all extracted samples and injected intermittently throughout the analysis run). All centroid data were Pareto scaled and analysed by unsupervised Principle Component Analysis (PCA) and supervised discriminatory analysis by Orthogonal Projections of Latent Structures-Discriminant Analysis (OPLS-DA). Unsupervised PCA models were generated at each sampling day to reveal potential relationships between treatment groups. Supervised analysis by OPLS-DA was performed to reveal potential markers of response to treatment in vaccinated calves compared to non-vaccinated calves at each sampling day. Robustness of final OPLS-DA discriminative models was assessed by setting a predictive model of each case in which 2/3 of the data (known treatment) was used to predict the remaining 1/3 (unknown treatment). Significance of the identified markers at all time points was determined using ANOVA with Bonferroni post-hoc test and non-parametric Mann Whitney for day 14 and 21 p.i. time points.

### Identification of plasma metabolite markers

The elemental composition of selected parent compounds was determined in MassLynx using both positive and negative mode data. Mass uncertainty was set to 3 mDa, odd and even electron state, carbon isotope filter of +/- 5% and elements included were C, H, O, N, P and S. Where applicable Na and K adduct elemental composition were determined with the respective element included in the analysis parameters. Elemental compositions were searched against PubChem and Chemspider online databases, and where possible Function 2 fragments were matched against Metlin, HMDB or Massbank databases. Where fragmentation spectra for the analyte in question was not available, *in silico* fragmentation was performed using Metfrag and Function 2 fragmentation data was validated against potential *in silico* fragments. Identified compounds were validated using mass spectrum and retention time relative to authentic analytical standards analysed under identical experimental conditions (S4 Fig). Pooled plasma samples and individual analytical standards (1 μM) were analysed under identical UPLC and mass spectrometric run conditions as utilized previously [22], and metabolite identities confirmed by matching retention time and Function 1 and Function 2 spectra (including low and high energy fragments and adducts). Putative annotations were acquired for compounds with spectral similarity to public spectral libraries (Metlin, HMCB or Massbank).

## Supporting information

S1 Table Inter-run quality control parameters for plasma analysed using Xevo G2 Qtof by RP-UPLC-MS/MS and HILIC-UPLC-MS/MS. For RP-UPLC-MS/MS analysis runs peaks corresponding to L-phenylalanine, L-tryptophan and Glycocholic acid were analysed and for HILIC-UPLC-MS/MS creatine, creatinine and L-valine were analysed. Average, min, max and Stdev for retention time and mass accuracy are reported.
(XLSX)

S2 Table Cross-validation of OPLS-DA models. Leave-one-out (LOO) cross validation was performed to assess the performance of the models generated. All technical replicated from a single biological sample were removed and an OPLS-DA model containing the remaining biological and technical replicates employed to predict class representation.
(XLSX)

S3 Table Selected AMRTPs of plasma metabolomic markers of response BPI3V challenge in vaccinated and non-vaccinated animals. Combined list of 383 AMRTPs selected using OPLS-DA at days 2, 6, 14 and 20 post-BPI3V infection filtered to exclude those with p < 0.05, FC < 1.5 and V.I.P. score < 1.
(XLSX)

S4 Table Processed Raw data employed for downstream metabolomic analysis. Markerlynx was employed for data extraction and processing. AMRTP levels are reported as peak height.
(XLSX)

S1 Fig BPI chromatogram of plasma from vaccinated and non-vaccinated calves analysed by RP-UPLC and HILIC-UPLC-MS/MS at day 6 p.i. (PDF)

S2 Fig Peak intensity of identified metabolite markers in individual animals throughout the study. Significant changes in metabolite peak intensity (height) are indicated NS = p > 0.05, * = p < 0.05, ** = p < 0.01, *** = p < 0.001.
(PDF)

S3 Fig Spectral profiles for N-(cyclohex-1-en-1-ylcarbonyl)glycine (NCG), Hexahydrohippurate (HHA) and N-Methylhippuric Acid (NMHA). (A) Low energy fragmentation and adduct formation spectra. (B) High energy fragmentation and adduct formation spectra.
(PDF)

S4 Fig Identification of DIVA metabolite markers in plasma using Waters Xevo G2 Qtof. Retention time matching, and MS spectrum of (A) biliverdin, (B) cholic acid, (C) hexahydrophippuric acid and (D) lysophosphatidylcholine standards with extracted ion chromatograms (low energy and high energy ramp) of DIVA metabolite markers.
(PDF)
