# Supplementary material for: DIVA metabolomics: Differentiating vaccination status following viral challenge using metabolomic profiles
Source: PLoS One. 2018 Apr 5;13(4):e0194488. doi: 10.1371/journal.pone.0194488 (PMC5886402; doi:10.1371/journal.pone.0194488)
Supplement: S1 Fig — (PDF) [file pone.0194488.s005.pdf]

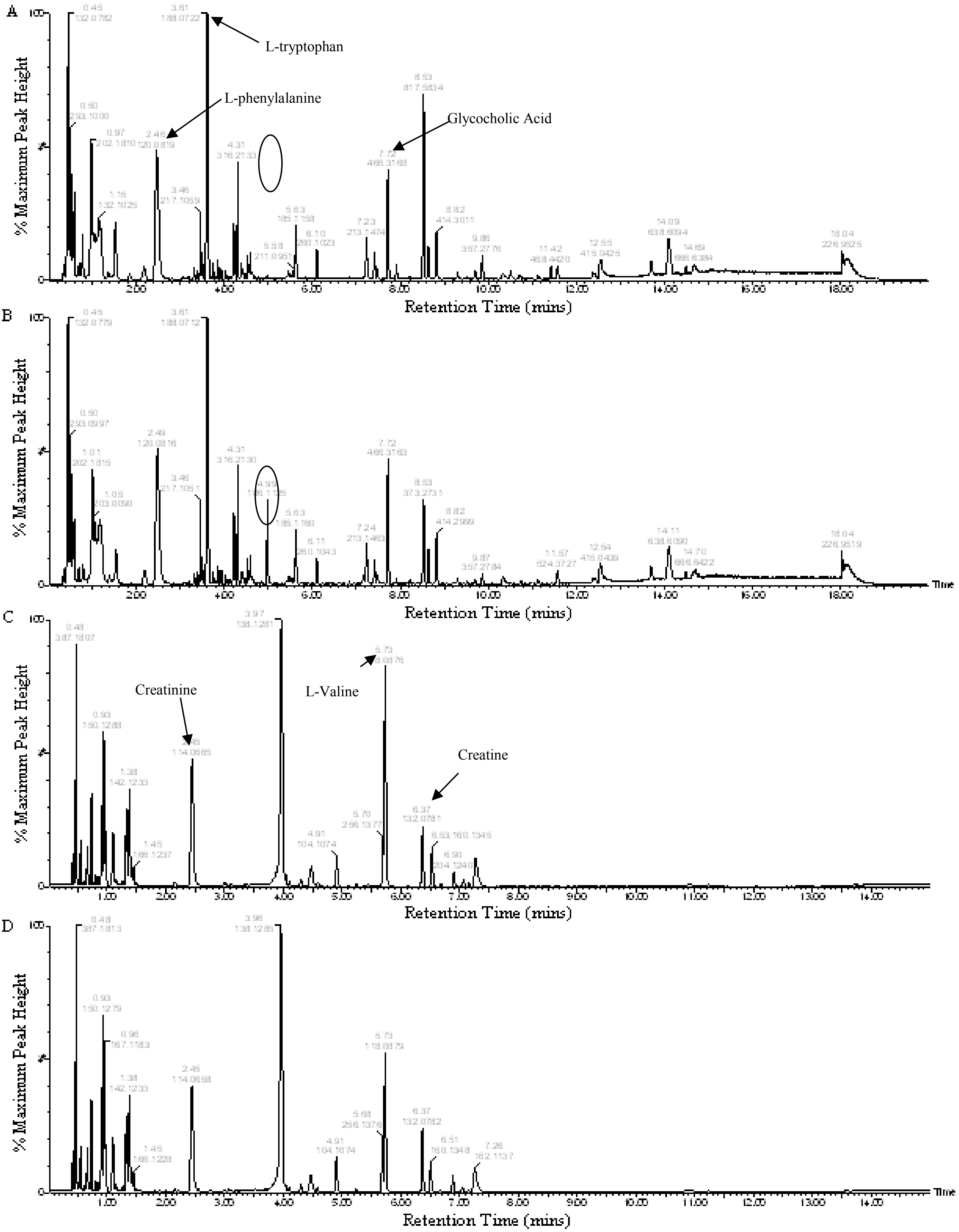

Figure 1: BPI chromatogram of plasma analysed by (A+B) RP-UPLC and (C+D) HILIC-UPLC-MS/MS (C+D) at day 6 p.i. For RP-UPLC-MS/MS intensity threshold is a maximum of 80,000 counts to reduce the influence of tryptophan (3.61 min) and observe a greater number of chromatographic peaks near the baseline. Plasma from non-vaccinated animals is indicated in figures A and C, and vaccinated calves in B and D. Peaks quality control standards for tryptophan, phenylalanine and glycocholic (RP-UPLC-MS/MS) acid creatinine, creatine and valine (HILIC-UPLS-MS/MS) are indicated.
