# Supplementary material for: DIVA metabolomics: Differentiating vaccination status following viral challenge using metabolomic profiles
Source: PLoS One. 2018 Apr 5;13(4):e0194488. doi: 10.1371/journal.pone.0194488 (PMC5886402; doi:10.1371/journal.pone.0194488)
Supplement: S2 Fig — Significant changes in metabolite peak intensity (height) are indicated NS = p > 0.05, * = p < 0.05, ** = p < 0.01, *** = p < 0.001. (PDF) [file pone.0194488.s006.pdf]

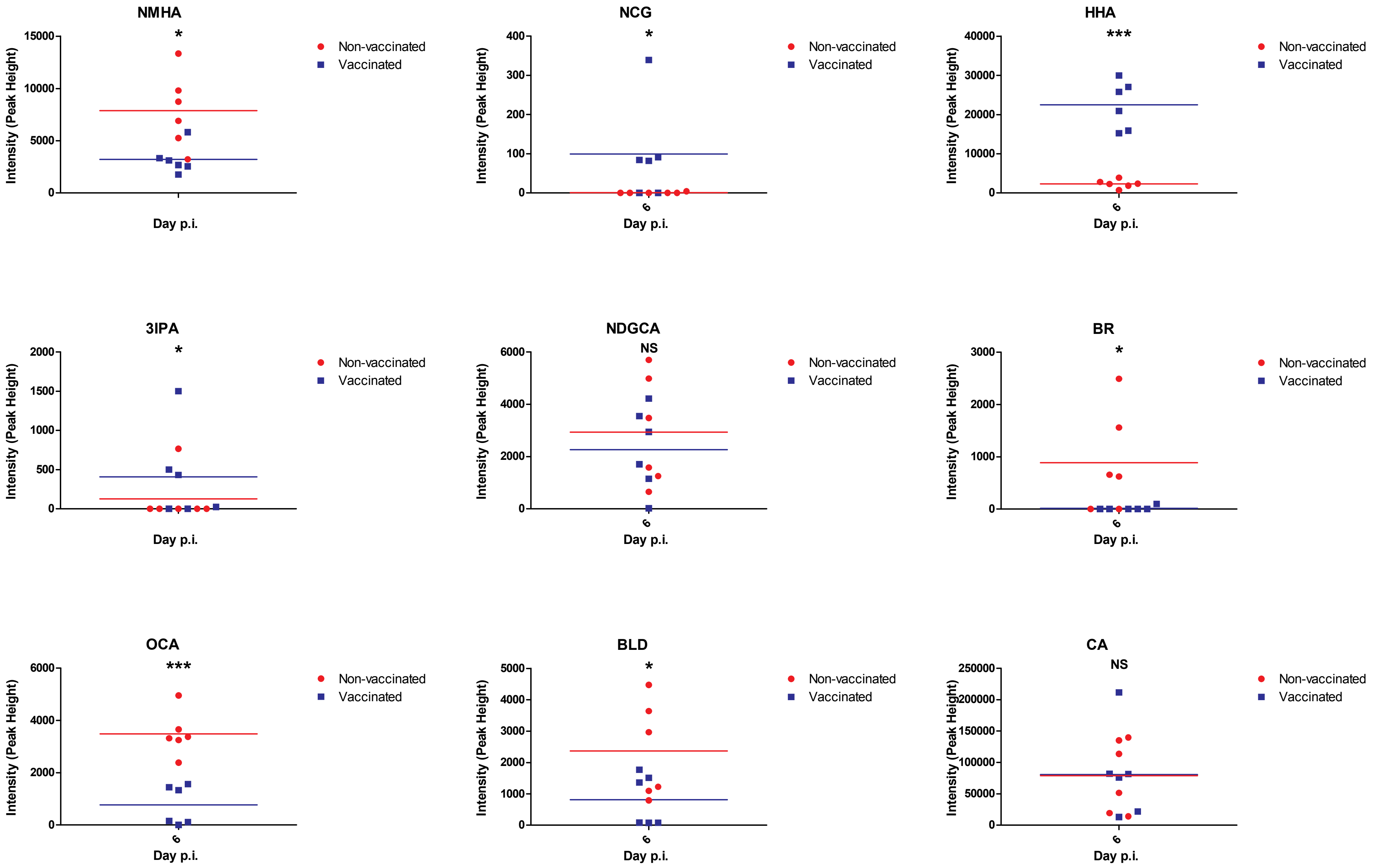

Figure 2. Peak intensity of identified metabolites in individual animals from analysis run 1 (day 6 p.i.). Significant changes in metabolite peak intensity (height) are indicated NS = p>0.05, * = p<0.05, ** = p<0.01, *** = p<0.001.

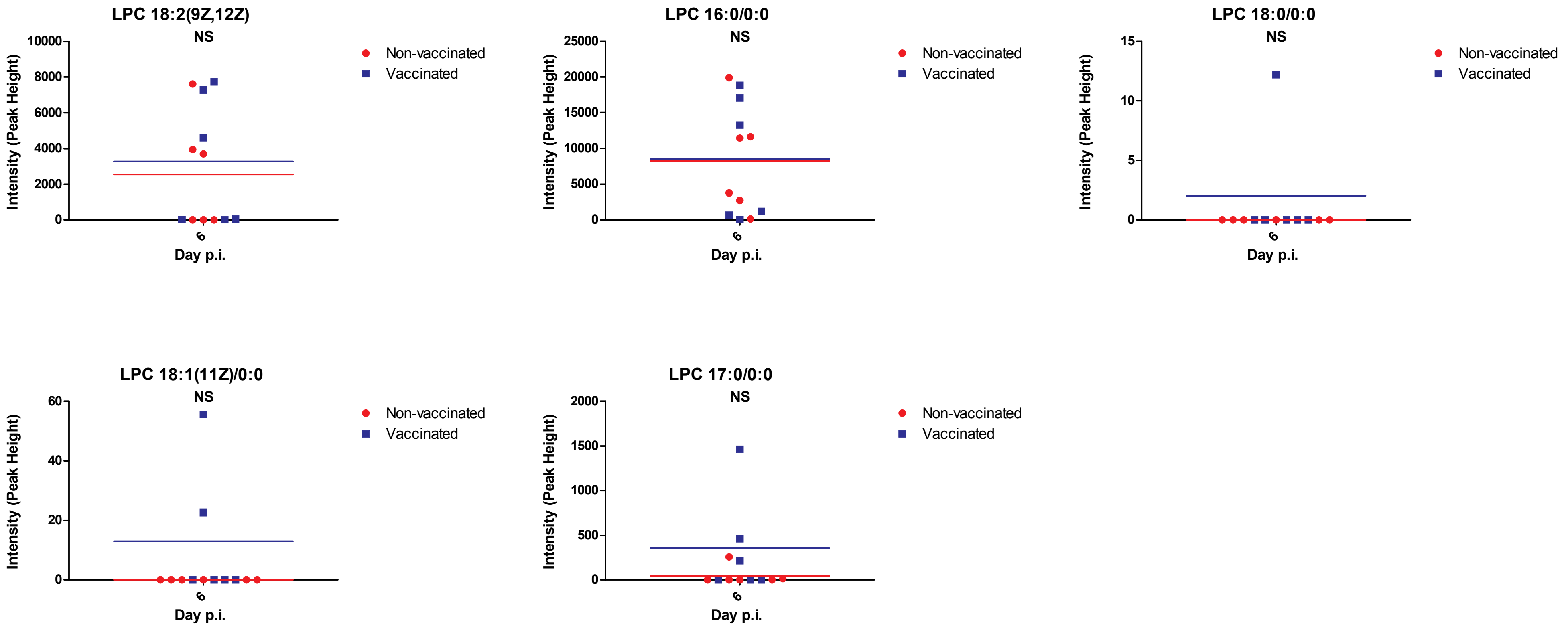

Figure 2. Peak intensity of identified metabolites in individual animals from analysis run 1 (day 6 p.i.). Significant changes in metabolite peak intensity (height) are indicated NS = $p>0.05$, * = $p<0.05$, ** = $p<0.01$, *** = $p<0.001$.

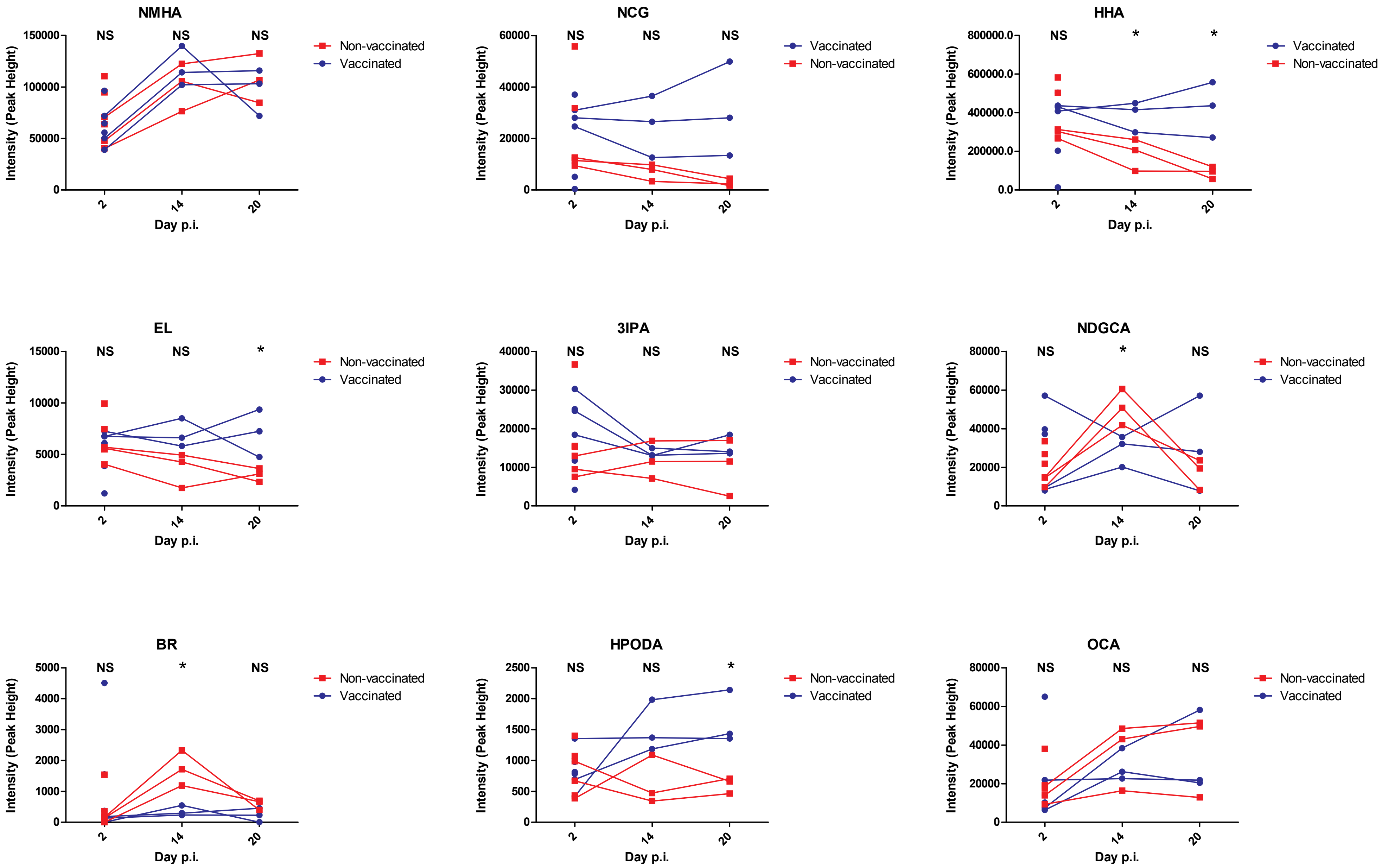

Figure 2. Peak intensity of identified metabolites in individual animals from analysis run 2 (days 2, 14 and 20 p.i.). Significant changes in metabolite peak intensity (height) are indicated NS = p>0.05, * = p<0.05, ** = p<0.01, *** = p<0.001. Peak intenensity for individual animals is connect across sampling days.

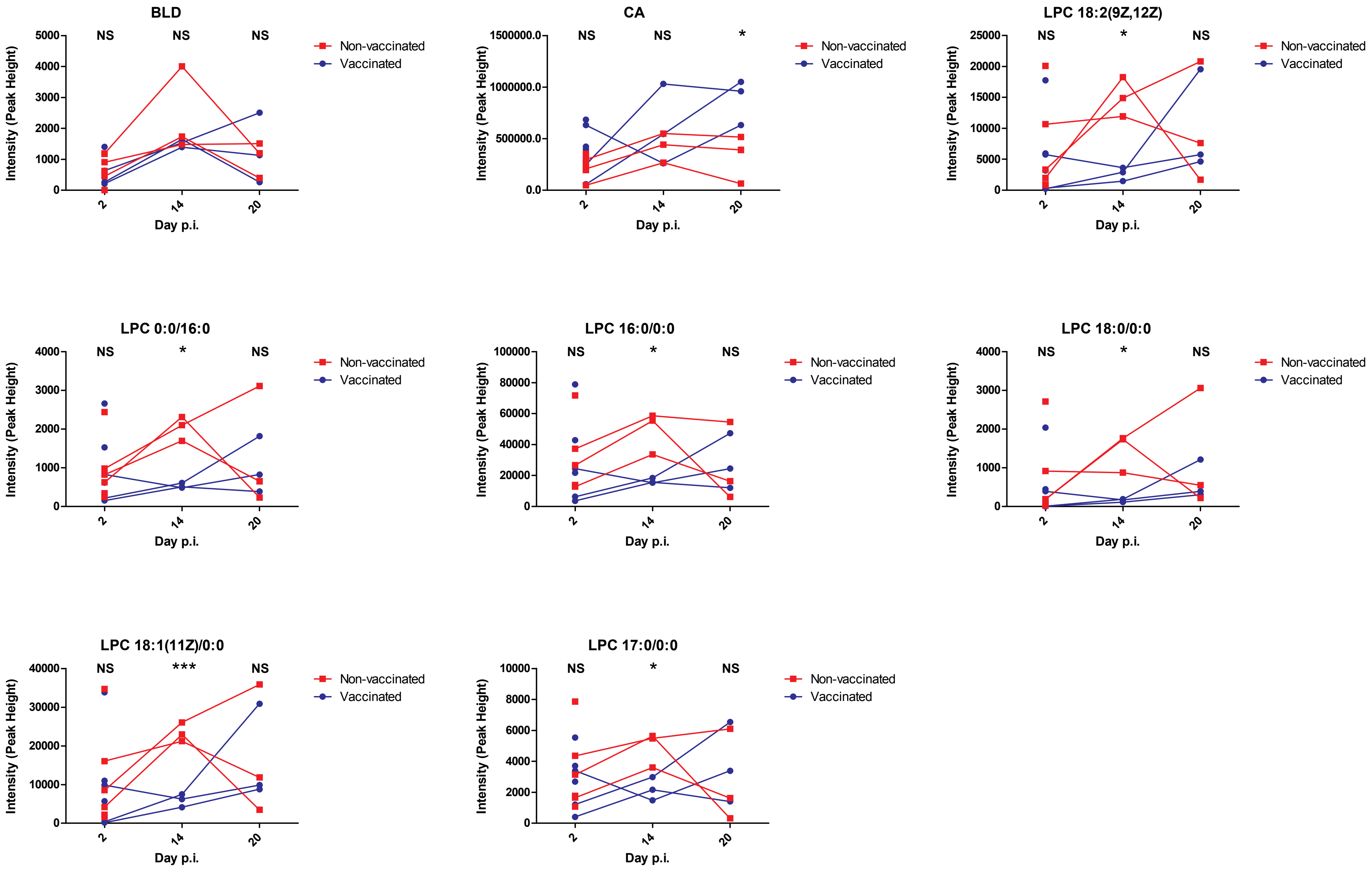

Figure 2. Peak intensity of identified metabolites in individual animals from analysis run 2 (days 2, 14 and 20 p.i.). Significant changes in metabolite peak intensity (height) are indicated NS = p>0.05, * = p<0.05, ** = p<0.01, *** = p<0.001. Peak intenensity for individual animals is connect across sampling days.
