# Supplementary material for: DIVA metabolomics: Differentiating vaccination status following viral challenge using metabolomic profiles
Source: PLoS One. 2018 Apr 5;13(4):e0194488. doi: 10.1371/journal.pone.0194488 (PMC5886402; doi:10.1371/journal.pone.0194488)
Supplement: S3 Fig — (A) Low energy fragmentation and adduct formation spectra. (B) High energy fragmentation and adduct formation spectra. (PDF) [file pone.0194488.s007.pdf]

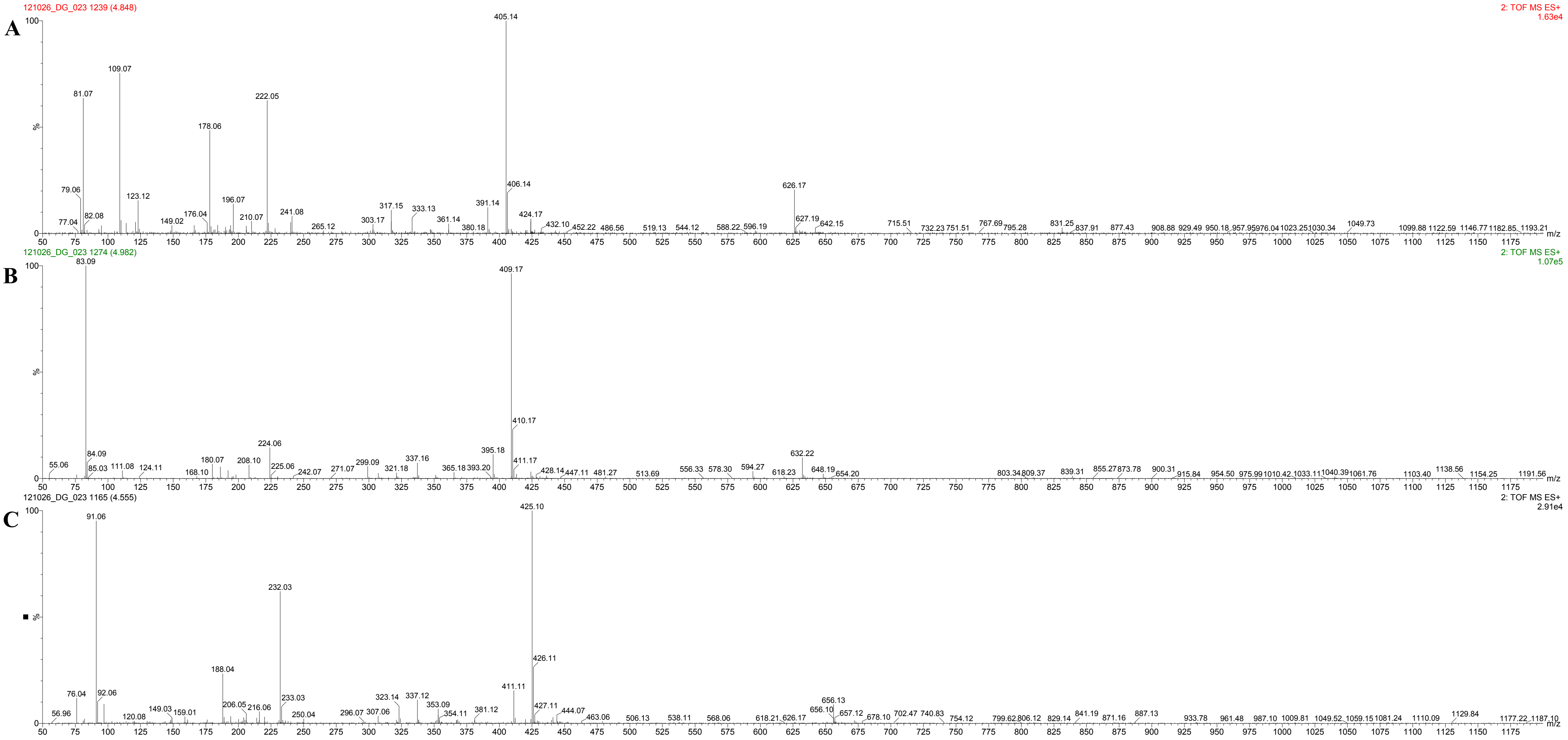

Figure 3A. Function 1 (low energy) fragmentation spectra of (A) N-(cyclohex-1-en-1-ylcarbonyl)glycine (NCG), (B) Hexahydrohippurate (HHA) and (C) N-Methylhippuric Acid (NMHA) in plasma.

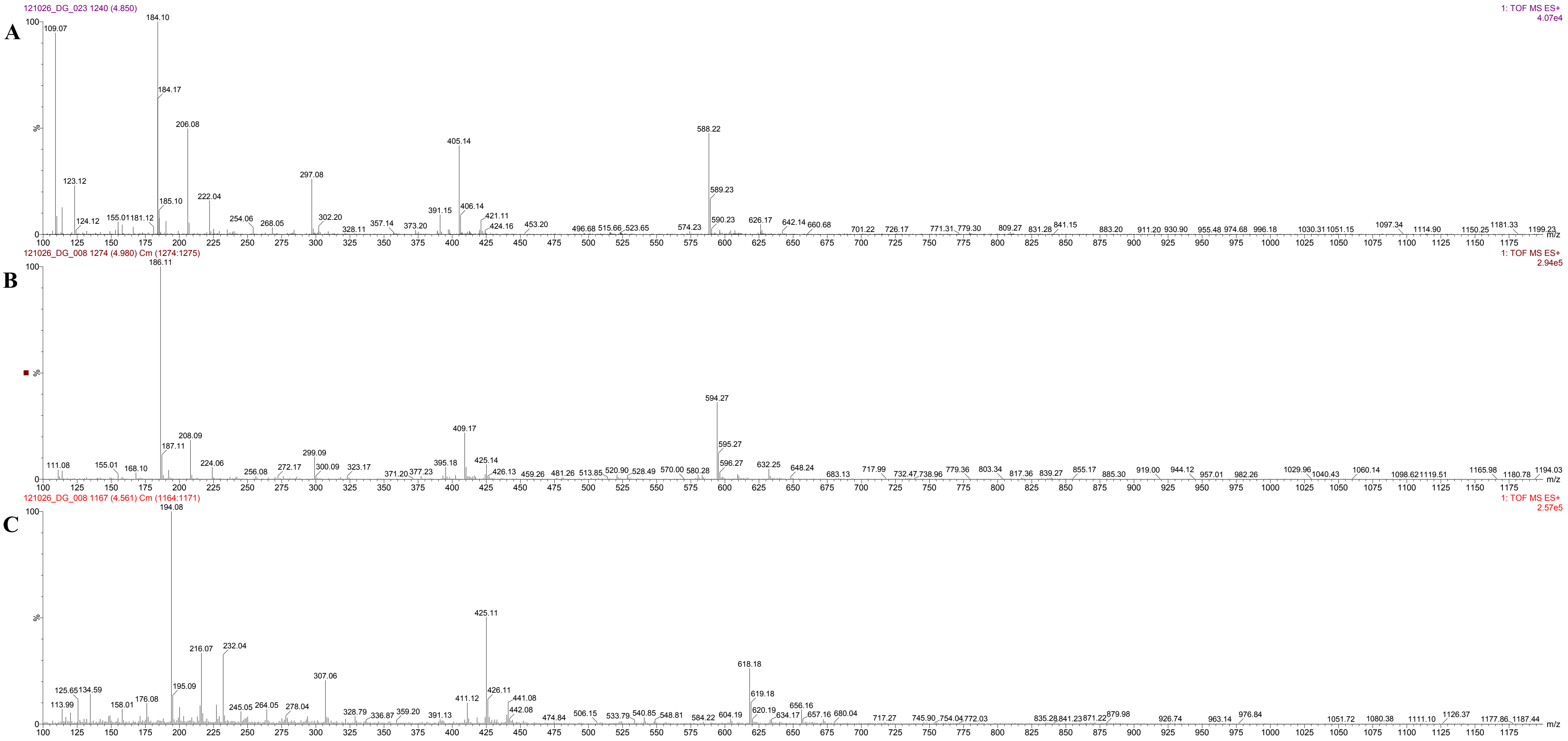

Figure 3B. Function 2 (high energy ramp) fragmentation spectra of (A) N-(cyclohex-1-en-1-ylcarbonyl)glycine (NCG), (B) Hexahydrohippurate (HHA) and (C) N-Methylhippuric Acid (NMHA) in plasma.
