# Supplementary material for: DIVA metabolomics: Differentiating vaccination status following viral challenge using metabolomic profiles
Source: PLoS One. 2018 Apr 5;13(4):e0194488. doi: 10.1371/journal.pone.0194488 (PMC5886402; doi:10.1371/journal.pone.0194488)
Supplement: S4 Fig — Retention time matching, and MS spectrum of (A) biliverdin, (B) cholic acid, (C) hexahydrophippuric acid and (D) lysophosphatidylcholine standards with extracted ion chromatograms (low energy and high energy ramp) of DIVA metabolite markers. (PDF) [file pone.0194488.s008.pdf]

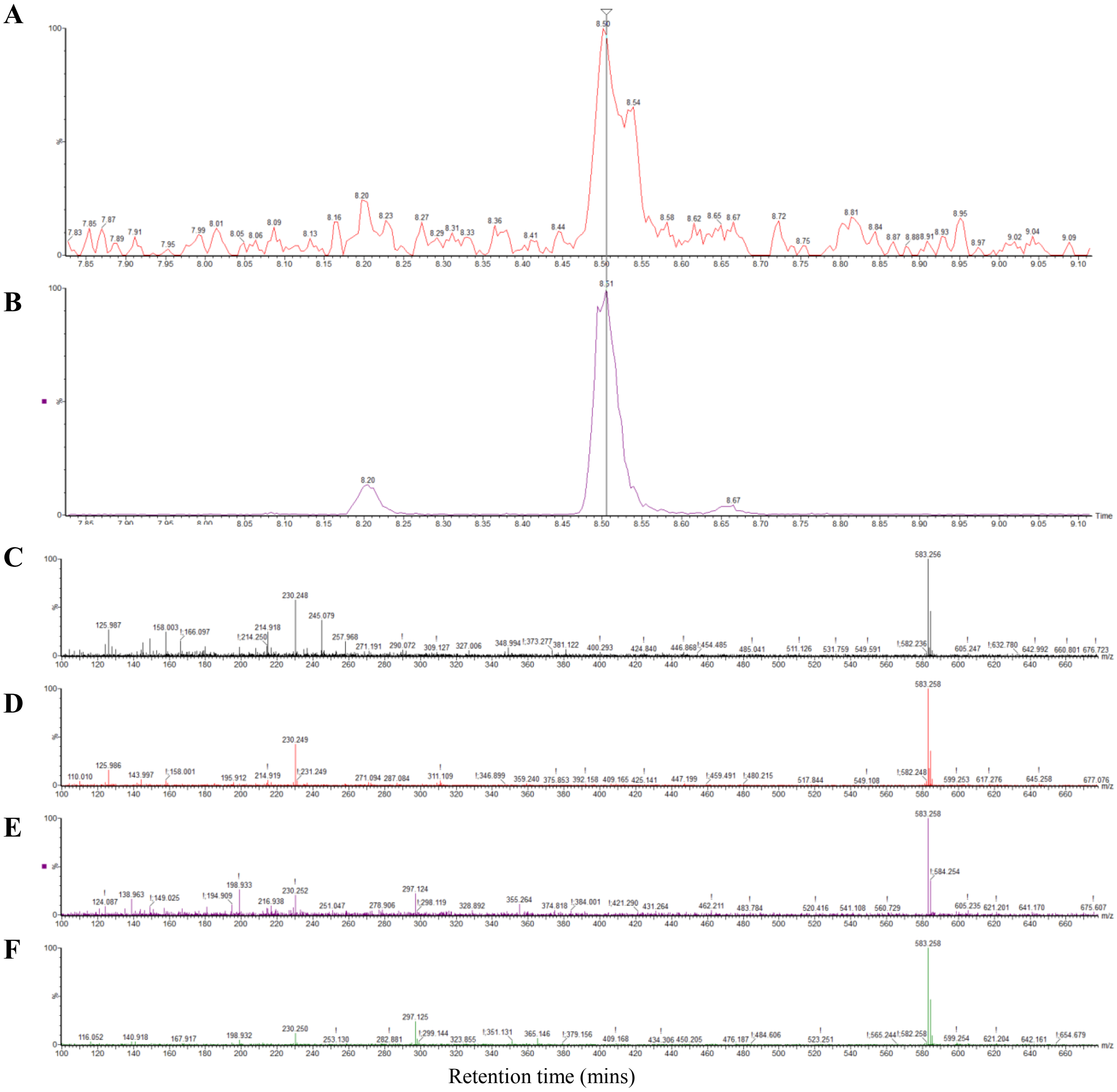

Figure 4A: Identification of Bilverdin (BLD) in plasma using Waters Xevo G2 Qtof. (A) Chromatographic peak of compound with mass/z 583.258 in plasma. (B) Chromatographic peak of 1M of BLD standard prepared in 0.1% formic acid, 99.9% acetonitrile. (C) Function 1 (low energy) fragmentation spectra of compound with mass/z 583.258 in plasma. (D) Function 1 (low energy) fragmentation spectra of BLD standard prepared in 0.1% formic acid, 99.9% acetonitrile. (E) Function 2 (high energy ramp) fragmentation spectra of compound with mass/z 583.258 in plasma. (F) Function 2 (high energy ramp) fragmentation spectra of BLD standard prepared in 0.1% formic acid, 99.9% acetonitrile.

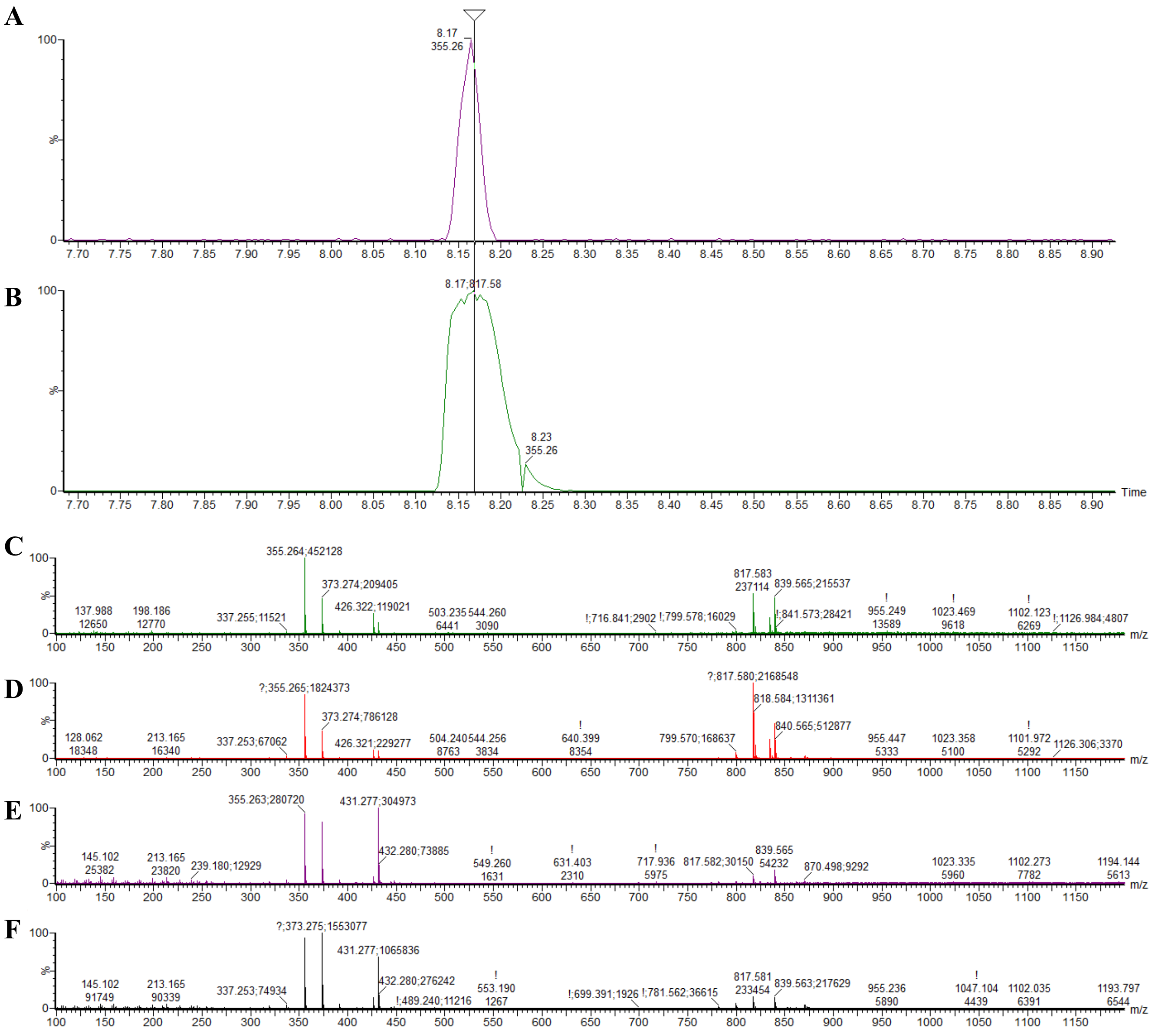

Retention time (mins)

Figure 4B: Identification of Cholic Acid (CA) in plasma using Waters Xevo G2 Qtof. (A) Chromatographic peak of compound with mass/z 817.583 in plasma. (B) Chromatographic peak of 1M of CA standard prepared in methanol. (C) Function 1 (low energy) fragmentation spectra of compound with mass/z 817.583 in plasma. (D) Function 1 (low energy) fragmentation spectra of CA standard prepared in methanol. (E) Function 2 (high energy ramp) fragmentation spectra of compound with mass/z 817.583 in plasma. (F) Function 2 (high energy ramp) fragmentation spectra of CA standard prepared in methanol.

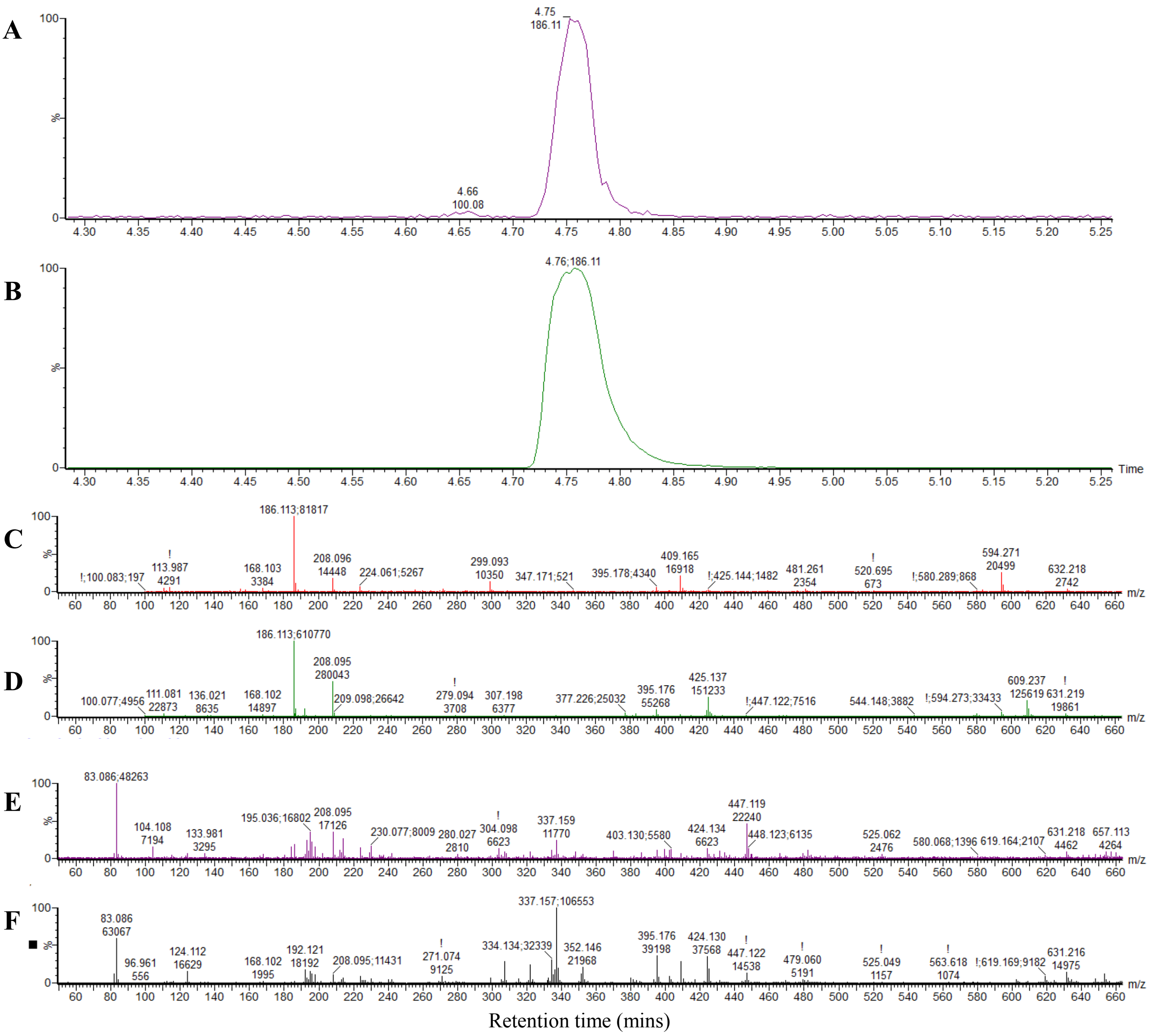

Figure 4C: Identification of Hexahydrohippuric Acid (HHA) in plasma using Waters Xevo G2 Qtof. (A) Chromatographic peak of compound with mass/z 186.113 in plasma. (B) Chromatographic peak of 1M of HHA standard prepared in methanol. (C) Function 1 (low energy) fragmentation spectra of compound with mass/z 186.113 in plasma. (D) Function 1 (low energy) fragmentation spectra of HHA standard prepared in methanol. (E) Function 2 (high energy ramp) fragmentation spectra of compound with mass/z 186.113 in plasma. (F) Function 2 (high energy ramp) fragmentation spectra of HHA standard prepared in methanol.

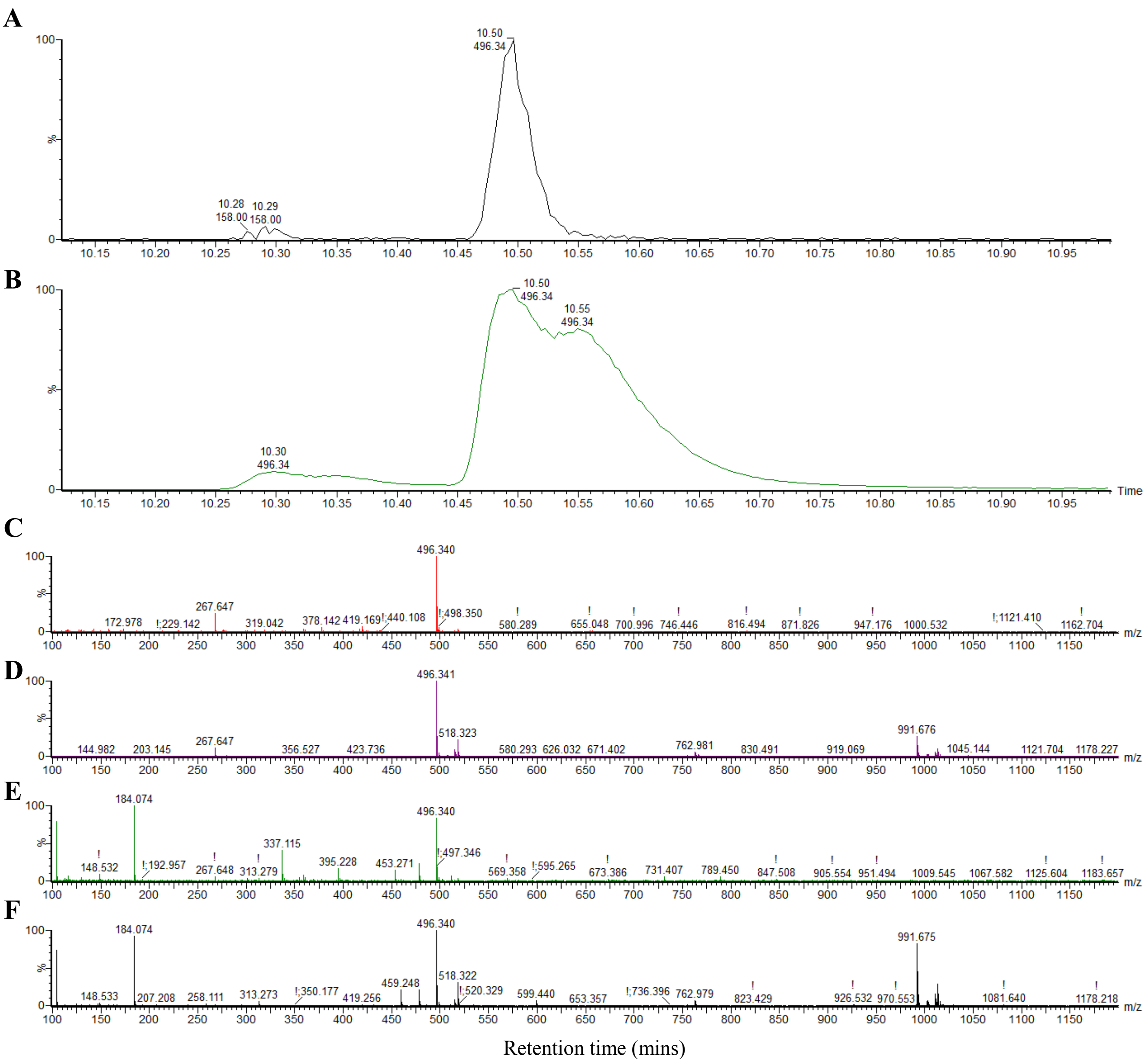

Figure 4D: Identification Lysophosphatidylcholine (LPC) in plasma using Waters Xevo G2 Qtof. (A) Chromatographic peak of compound with mass/z 496.340 in plasma. (B) Chromatographic peak of 1M of LPC standard prepared in methanol. (C) Function 1 (low energy) fragmentation spectra of compound with mass/z 496.340 in plasma. (D) Function 1 (low energy) fragmentation spectra of LPC standard prepared in methanol. (E) Function 2 (high energy ramp) fragmentation spectra of compound with mass/z 496.340 in plasma. (F) Function 2 (high energy ramp) fragmentation spectra of LPC standard prepared in methanol.
